# Genome-Wide Characterization, Evolution, and Expression Analysis of the Leucine-Rich Repeat Receptor-Like Protein Kinase (LRR-RLK) Gene Family in *Medicago truncatula*

**DOI:** 10.3390/life10090176

**Published:** 2020-09-04

**Authors:** Juan Meng, Jie Yang, Mengdi Peng, Xiaolin Liu, Hengbin He

**Affiliations:** Beijing Key Laboratory of Ornamental Plants Germplasm Innovation and Molecular Breeding, National Engineering Research Center for Floriculture, Beijing Laboratory of Urban and Rural Ecological Environment, School of Landscape Architecture, Beijing Forestry University, Beijing 100083, China; juanmeng@bjfu.edu.cn (J.M.); jieyang@bjfu.edu.cn (J.Y.); mengdi2020@bjfu.edu.cn (M.P.); liuxiaolin@bjfu.edu.cn (X.L.)

**Keywords:** *M. truncatula*, leucine-rich repeat receptor-like kinase (LRR-RLKs), phylogenetic analysis, evolutionary analysis, expression profiling

## Abstract

Leucine-rich repeat receptor-like kinases (LRR-RLKs) constitute the largest subfamily of receptor-like kinases (RLKs) in plants. They play roles in plant growth and developmental and physiological processes, but less is known about the functions of LRR-RLKs in *Medicago truncatula*. Our genome-wide analysis revealed 329 *LRR-RLK* genes in the *M.* *truncatula* genome. Phylogenetic and classification analysis suggested that these genes could be classified into 15 groups and 24 subgroups. A total of 321 genes were mapped onto all chromosomes, and 23 tandem duplications (TDs) involving 56 genes were distributed on each chromosome except 4. Twenty-seven *M.* *truncatula* LRR-RLK segmental duplication gene pairs were colinearly related. The exon/intron organization, motif composition and arrangements were relatively conserved among members of the same groups or subgroups. Using publicly available RNAseq data and quantitative real-time polymerase chain reaction (qRT-PCR), expression profiling suggested that *LRR-RLKs* were differentially expressed among different tissues, while some were expressed specifically in the roots and nodules. The expression of *LRR-RLKs* in A17 and 4 nodule mutants under rhizobial infection showed that 36 *LRR-RKLs* were highly upregulated in the *sickle* (*skl*) mutant [an ethylene (ET)-insensitive, Nod factor-hypersensitive mutant] after 12 h of rhizobium inoculation. Among these *LRR-RLKs*, six genes were also expressed specifically in the roots and nodules, which might be specific to the Nod factor and involved in autoregulation of the nodulation signal. Our results provide information on the *LRR-RLK* gene family in *M. truncatula* and serve as a guide for functional research of the *LRR-RLKs*.

## 1. Background

Transmembrane receptor kinases play critical roles in both animal and plant signaling pathways involved in the regulation of growth, development, differentiation, cell death, and defense responses to pathogens [1,2,3]. Previous studies have shown that receptor-like kinases (RLKs) in plants are similar to receptor tyrosine kinases (RTKs) in animals [4]. Among animal kinase sequences, the Drosophila Pelle and related cytoplasmic kinases fall within the plant RLK clade, which has been defined as the RLK/Pelle family [5,6,7]. RLKs are classified into two broad categories on the basis of whether their transmembrane proteins have extracellular domains—receptor-like cytoplasmic kinases (RLCKs) and receptor-like protein kinases (RLKs) [6,7,8,9]. A typical RLK contains an extracellular domain, transmembrane domain and intracellular kinase domain; RLKs constitute one of the largest gene families in flowering plants such as *Arabidopsis*. The first plant RLK gene was found and cloned in maize and was shown to encode a receptor kinase protein [10]. In *Arabidopsis* alone, it has been reported that there are at least 610 RLK members, representing nearly 2.5% of *Arabidopsis* protein-coding genes [1]. Surprisingly, the rice (*Oryza sativa*) genome contains more than 1000 members, with nearly twice as many RLK/Pelle members as *Arabidopsis* has [2,11,12]. Depending on their type of extracellular domain, RLKs can be classified into different at least 17 subgroups [6,7]. The largest subgroup is the leucine-rich repeat (LRR) RLK family, whose members contain an extracellular LRR domain to recognize and receive external signals. A previous study indicated that an LRR-RLK protein contain at least one LRR domain, generally [12]. An LRR, also named the LRR domain, is composed of 20–30 repeating amino acid (aa) stretches that are unusually enrich in the hydrophobic aa leucine. Each LRR domain is composed of a beta-alpha unit and there are more than 13 families of LRR domains in Pham database [13,14]. Each repeated unit typically has a β-strand-turn-α-helix structure, which is a protein structural motif that forms an α/β horseshoe fold [13,14]. LRRs are frequently involved in the formation of protein–protein interactions [15,16].

In *Arabidopsis*, the leucine-rich repeat receptor-like kinase (LRR-RLK) family is a large family with more than 200 members [1,6,7,8,17,18]. Its members can be divided into 12–15 groups and 23 subgroups according to the number and arrangement of LRR motifs within the extracellular domains [1,6,7,8]. In the limited cases where a functional role has been purported for plant LRR-RLKs, they have been shown to be involved in a wide range of plant growth, developmental and physiological processes; hormone perception; brassinosteroid (BR) signaling; and defense responses to bacterial pathogens [8,19,20,21]. BRs are natural growth-promoting products found at low levels in pollen, seeds, and young vegetative tissues throughout the plant kingdom [22]. BRASSINOSTEROID-INSENSITIVE 1 (BRI1) is involved in BR signal transduction. The extracellular domain of BRI1 contains 25 tandem LRRs that resemble repeats found in animal hormone receptors [23,24]. BRI1-associated receptor kinase 1 (BAK1), another LRR-RLK, has been suggested to interact with BRI1 to activate its kinase activity through transphosphorylation. These proteins function together to mediate plant steroid signaling [25]. In *Arabidopsis*, the BAK1-interacting receptor-like kinase 1(BIR1) and BAK1 protein kinases also interact with the calcium-dependent phospholipid-binding protein BON1 in the modulation of temperature-dependent plant growth and cell death [26]. ERECTA (ER) is another important LRR-RLK protein belonging to the ERECTA family (ERF), which also contains two other members—ERECTA LIKE 1 (ERL1) and ERECTA LIKE 2 (ERL2). These proteins are involved in regulating cotyledons, organ growth, flower development and biotic/abiotic stress responses in *Arabidopsis* [27,28,29]. BRI1 KINASE INHIBITOR1 (BKI1) functions as a common suppressor of the BRI1 and ER signaling pathways, inhibiting ER kinase activity to regulate plant architecture [30].

As research has intensified, an increasing number of LRR-RLK functions have been gradually discovered. Previous studies have shown that CLAVATA (CLV) and Receptor-like protein kinase 2 (RPK2) are important genes involved in meristem regulation [31,32,33,34,35,36]. RGF1 INSENSITIVE 1 to 5, which compose a group of LRR-RLKs, are essential for the perception of root meristem growth factor 1 in *Arabidopsis* [37]. EXCESS MICROSPOROCYTES 1 (AtEMS1) and SOMATIC EMBRYOGENESIS RECEPTOR-LIKE KINASE 1 (GhSERK1) control somatic embryogenesis and pollen production in *Arabidopsis* and *Gossypium*, respectively [38,39]. Moreover, LRR-RLKs function in both plants and animals to detect specific pathogen peptides, after which Pelle-family kinases relay signals from those receptors [40]. Flagellin-sensitive 2 (FLS2), an LRR-RLK, recognizes a conserved 22 aa N-terminal sequence of the bacterial flagellin protein (flg22), whose LRR domain perceives flg22 and rapidly recruits another LRR-RLK BAK1 [41]. FLS also interacts with the receptor-like cytoplasmic kinases (RLCK) BOTRYTIS-INDUCED KINASE 1(BIK1) and binds to the immune receptor complex directly during perception of the bacterial elicitor flagellin in *Arabidopsis* [42]. In addition, EFR is another LRR-RLK that can perceive the bacterial EF-Tu protein in *Arabidopsis*; this activity is separate from that of the flagellin receptor FLS2 but elicits a set of highly similar defense responses [43]. In view of their important functions in plants, members of the LRR-RLK gene family have been identified in poplar, rice, soybean, five Rosaceae species (strawberry, apple, white pear plum, peach), two *Citrus* species, and *Amborella trichopoda* [12,44,45,46,47,48].

*Medicago truncatula* is a typical model plant legume species and is among the various research materials associated with nodule symbiosis [49,50]. It was found that the LRR receptor kinase (nodulation receptor kinase) DOES NOT MAKE INFECTIONS 2 (DMI2) plays an important role in the reception and transmission of signals involved in symbiosis [51,52,53,54]. In this study, a genome-wide search for LRR-RLK genes was performed in *M. truncatula*, and a total of 329 *M. truncatula* LRR-RLKs (MtLRR-RLKs) were identified. We aimed to determine the phylogenetic relationships of these genes with those of *Arabidopsis* and soybean, as well as their chromosomal locations, structure, tandem and segmental duplication events, and expression, and predict related cis-acting elements, which should provide genome-level insights into LRR-RLK genes.

## 2. Materials and Methods

### 2.1. Arabidopsis, Soybean LRR-RLK Family and M. truncatula Genomic Resources

A previous study revealed 225 and 467 LRR-RLK genes in *Arabidopsis* and soybean based on the sequence similarity of kinase domains [8,45,46]. The genomic resources of putative AtLRR-RLK and GmLRR-RLK family members were acquired from The Arabidopsis Information Resource (TAIR) database 10 and from Glycine max Wm82.a2.v1 in the Phytozome 12.1 database (https://phytozome.jgi.doe.gov/pz/portal.html), respectively [55]. The genomic sequences, coding sequences and database peptide sequences of all annotated *M. truncatula* genes were obtained from *Medicago truncatula Mt4.0v1* genomic sequences in the Phytozome 12.1 database (https://phytozome.jgi.doe.gov/pz/portal.html) [55].

### 2.2. Identification of LRR-RLK Genes in the M. truncatula Genome

As previously known, LRR-RLKs contain two typical domains—extracellular LRR domains and the intracellular kinase domain. To identify LRR-RLK genes, the Hidden Markov Model (HMM) search method was used [56]. Putative LRRs were initially obtained by searching HMMs of the leucine-rich repeat clan [LRR1 (PF00560), LRR5 (PF13306), LRR6 (PF13516), LRR8 (PF13855) and LRRNT-2 (PF08263)], obtained from the Pfam database 32.0 (http://pfam.xfam.org) [57], using HMMER 3.1 software in Linux. The sequences were screened again with an E-value that was less than 0.001, and only the first transcript was used. Furthermore, these sequences were filtered by their description and functional annotation via the Phytozome 12 database (https://phytozome.jgi.doe.gov/pz/portal.html#!search?show=KEYWORD&method=Org_Mtruncatula), followed by analysis with SMART (http://smart.embl-heidelberg.de) [58], the Pfam database (http://pfam.xfam.org/) and the NCBI Conserved Domains Database (CDD) (https://www.ncbi.nlm.nih.gov/cdd) to ensure the presence of LRR and kinase domains. To prevent members from being missed, we performed a BLASTp search against the aa sequences of all annotated *M. truncatula* proteins with a homologous LRR-RLK (AtG09970) in *Arabidopsis thaliana*. The members containing at least the LRR or kinase domain were then filtered and removed according to the same method as that used above. Finally, after repeated alignments with known *Arabidopsis* and soybean LRR-RLK family members, a phylogenetic tree was constructed, and inaccurate sequences were deleted.

### 2.3. Multiple Sequence Alignments and Phylogenetic Tree Analysis

Multiple sequence alignments were performed by using ClustalW and Muscle in MEGA 7.0 [59] with full-length aa sequences, which were transformed into coding sequences by MEGA 7.0. Unrooted phylogenetic trees were constructed for *M. truncatula LRR-RLK*s alone or for *M. truncatula*/soybean/*Arabidopsis* together with the neighbor-joining (NJ) method [60]. The nodes were tested by bootstrap analysis with 1000 replicates, and the tree with the highest likelihood was selected for further analysis. The evolutionary distances were computed using the *p*-distance method [3] and are expressed in units of numbers of aa differences per site. iTols (https://itol.embl.de/itol.cgi) [61], EvolView website (http://www.evolgenius.info/evolview) and AI software were then used to modify the phylogenetic tree.

### 2.4. Gene Structure and Protein Conserved Motif Analysis

The position of the mRNA, the position and number of introns and exons, and the UTRs were extracted as part of a batch system from the genomic annotation file in Linux. Combined with the information from the phylogenetic tree (*nwk* file), the exon–intron structures of the *MtLRR-RLK* genes were determined on the Gene Structure Display Server (GSDS) v2.0 website (http://gsds.cbi.pku.edu.cn/index.php). The conserved motifs of each *MtLRR-RLK* protein were predicted with MEME v4.12.0 [62], and figures were drawn by TBtools and AI software.

### 2.5. Chromosomal Location Analysis

To locate the *M. truncatula LRR-RLK* genes on the chromosomes, the locations of the *MtLRR-RLK*s and chromosome length information were acquired from the gff3 genomic annotation file in Linux. The *MtLRR-RLK* genes were mapped onto *M. truncatula* chromosomes based on their physical positions. Chromosomal location figures were drawn with Gene-map v2.0 (http://mg2c.iask.in/mg2c_v2.0.).

### 2.6. Duplication and Synteny Analysis

TDs were characterized as multiple members of this gene family occurring within neighboring intergenic regions [45]. The segmental duplicated genes of *MtLRR-RLK*s were identified by MCScanX [63] and CIRCOS [64] by the use of genomic protein sequences that were queried against themselves via BLAST. The synteny of the *MtLRR-RLK*s from different genomes, including *Arabidopsis* and soybean, was mapped by MCScanX, with collinearity determined in Linux as well. Tandem and segmental duplications were identified by MCScanX, and the *Ks* and *Ka* values for duplicated gene pairs were calculated based on the coding sequence alignments using KaKs_Calculator v2.0 [65]. The divergence times of the duplicated gene pairs were estimated by their synonymous mutation rate substitutions per synonymous site per year as follows—T = Ks/2γ (γ = 1.5 × 10^−8^), where γ is the rate of divergence for nuclear genes of dicotyledonous plants [66].

### 2.7. Analysis of Cis-Acting Elements within MtLRR-RLK Gene Promoter

The upstream sequences (2.0 kb) of the *MtLRR-RLK* genomic DNA were retrieved from the genomic sequence data in Linux and then submitted to the PlantCARE database (http://bioinformatics.psb.ugent.be/webtools/plantcare/html/) [67] to analyze the cis-acting elements. We ultimately selected 20 elements, including those induced by hormones, such as ABA-responsive, ET-responsive, stress-responsive elements; the stress-responsive elements included elements that responded to defense, low temperature and light. The GSDS v2.0 website (http://gsds.cbi.pku.edu.cn/index.php) was used to construct the map.

### 2.8. Expression Analysis

#### 2.8.1. Expression Analysis of MtLRR-RLK Genes in Different Tissues

The *M. truncatula* PLEX Experiment Data were downloaded from the Noble website [68]; https://mtgea.noble.org/v3/). Twelve different data sets were selected, which included data from 9 different tissues and data from root nodules at 3 different developmental times. On the website, the coding sequences were aligned in the expression database to determine the correspondence between the transcript gene number and chip number. The MtLRR-RLK expression data were subsequently obtained by the corresponding chip number, and then the mean of the three repeated expressions was used to map the heatmap in OmicShare via (Http://www.omicshare.com/tools/).

#### 2.8.2. Expression Analysis of MtLRR-RLK Genes under Rhizobial Infection

The expression data in response to rhizobial *Sinorhizobium medicae* infection were acquired by querying the gene expression RNAseq database (http://pages.discovery.wisc.edu/~sroy/Medicago_symbiosis_transcriptome/query.php), including that of WT and mutants with no or decreased Nod factor sensitivity [i.e., Nod factor sensitivity, Nodulation factor perception (*nfp*) and lysine motif domain-containing receptor-like kinase 3 (*lyk3*)] and an ET-insensitive and Nod factor-hypersensitive mutant (*sickle*, *skl*) [69]. Heatmaps were generated in OmicShare via (Http://www.omicshare.com/tools/) the means of three repeat expression data.

### 2.9. Plant Material, Treatment and qRT-PCR Analysis

*M. truncatula* seeds were scarified, germinated and grown in aeroponic tanks (caissons) or pots under the long day conditions—light/dark photoperiod, 16/8 h at temperature, 21 °C; humidity, 75%; light intensity (Photosynthetically Active Radiations, PAR), 300 μmol·m^−2^·s^−1^(HQL 400 De Luxe mercury vapor bulbs, Osram, 24,600 lux) [69]; Five *M. truncatula* genotypes, wild-type cv Jemalong A17, *nfp* (C31; [70]), *lyk3* (*hcl-1*, B56; [71]), *skl* (*skl1-1*; [72]), and *sunn* [73], were used.

Five-day-old plantlets were inoculated with *Sinorhizobium medicae* ABS7M. *S. medicae* ABS7M, grown in TY medium supplemented with 6 mM·L^−1^ calcium chloride and 10 μg·mL^−1^ tetracycline at 28 °C for 48 h. The culture was washed three times and finally resuspended in 10 mL sterile distilled water to an OD600 of 1.0, which was used to inoculate aeroponic caissons containing 10 L of low-nitrogen aeroponic medium [55]. Root samples were harvested at 0, 3, 6, 12, 24, and 48 hpi. Three independent biological replicates per time point and genotype were collected and immediately frozen in liquid nitrogen for RNA isolation.

Total RNA was isolated from the indicated tissues using Qiagen RNeasy kits (Qiagen). cDNA template was generated from equivalent quantities of RNA using a Quantitect Reverse transcription kit (Qiagen). Quantitative real-time polymerase chain reaction (qRT-PCR) was performed on an ABI 7500 Fast Real Time System (Applied Biosystems) using Power SYBR Green PCR Master Mix (Applied Biosystems), and the primers used are listed in Table S11. The PCR program consisted of an initial denaturation step (20 s at 50 °C) and a polymerase activation step of 10 min at 95 °C, followed by 40 cycles of 15s at 95 °C and 1 min at 60 °C. The relative expression level of gene was calculated using the ΔΔCt method and normalized using ubiquitin carrier protein (Medtr3g062450/TC17644) mRNA [69].

## 3. Results

### 3.1. Genome-Wide Identification and Conformation of LRR-RLK Genes in M. truncatula

To identify the members of the LRR-RLK gene family in the *M*. *truncatula* genome, hidden Markov models (HMMs) of the LRR domain in the Pfam database were used to query the whole genome. More than 1300 sequences with LRR domains were identified via HMM searches (Table S1). All the protein sequences were then submitted to the JGI, SMART, NCBI, and Pfam databases to annotate the domain structure. Only candidates containing at least one LRR domain and a kinase domain were considered “true” LRR-RLKs [45]. In total, 266 *MtLRR-RLK* sequences were ultimately obtained. To avoid the loss of members, we used typical LRR-RLK genes to query *M. truncatula* protein sequences and identified approximately 2000 sequences (Table S1). Finally, 71 additional sequences were obtained through the above method. After comparing these sequences with the LRR-RLK genes of *Arabidopsis* and soybean, we deleted 8 inaccurate sequences on the basis of the phylogenetic tree, and we ultimately identified 329 putative *LRR-RLK* genes in the *M*. *truncatula* genome (Table S1). All the identified LRR-RLK peptides ranged from 317 to 2123 aa in length, and most of them had transmembrane domains. Among them, 2 members (Medtr5g087360 and Medtr1g040615) had two kinase domains. Detailed information on the specific screening process, number of family members, subfamily information, aa size, molecular weight, and types as well as positions of conserved domains can be found in Table S1.

### 3.2. Phylogenetic Analysis and Classification of LRR-RLKs in Arabidopsis, M. truncatula and Soybean

**Figure 1:**
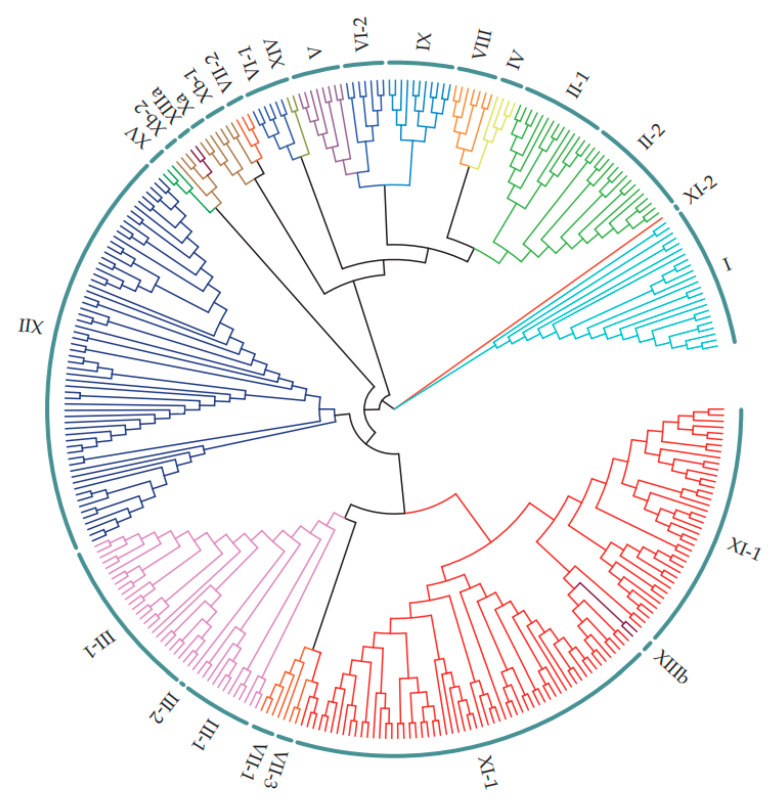
Phylogenetic analysis of LRR-RLKs retrieved in *M. truncatula*. The coding sequences for 329 MtLRR-RLKs were aligned by clusterW and the phylogenetic tree was constructed using MEGA 7.0 by the neighbor-joining method with 1000 bootstrap replicates. All MtLRR-RLKs were classified into 15 distinct groups and 24 subgroups based on the nomenclature of Arabidopsis LRR-RLKs (from I to XV) and were distinguished by different colors.

To investigate the evolutionary relationships of the LRR-RLK family members, we used MEGA software to construct a series of phylogenetic trees of all family members from *M*. *truncatula*, *Arabidopsis* and soybean [45]. The detailed information is listed in Figures S1 and S2. The members of *M*. *truncatula* essentially clustered according to the subgroups of LRR-RLK genes in *Arabidopsis* classified into 15 groups and 23 subgroups [8]. The 329 putative MtLRR-RLKs were classified into 15 groups and 24 subgroups (Table 1, Figure 1). The number of each subgroup member from *Arabidopsis*, *M*. *truncatula* and soybean was quantified, the sums of which are listed in Table 1. Most of the MtLRR-RLK members were classified according to the nomenclature of the *Arabidopsis* homologues within the same group (Table 1, Figure S2). In most subgroups, there were approximately twice as many members in soybean compared with *M*. *truncatula*, and each species had a cluster of members within a certain subgroup. For example, up to 50 members of AtLRR-RLK genes clustered in LRR-I; and 46 clustered in LRR- III-1. However, most MtLRR-RLK genes (99, the greatest number) clustered in LRR-XI-1, followed by LRR-XII (65) (Table 1 and Table S1, Figures S1 and S2). In the LRR-XI-1 subfamily, which contained 123 members from soybean and 99 members from *M*. *truncatula*, soybean and *M. truncatula* presented the most consistent clustering in this branch. Notably, in the LRR-XII subfamily, *M*. *truncatula* contained 65 members, which was more than twice the number in soybean, and there was separate and concentrated clustering.

Notably, the LRR-I group is classified into 2 subgroups in *Arabidopsis*, while in this study, LRR-I was considered only one group according to the nodes (Table 1). The LRR-II group of *M*. *truncatula* was classified into 2 subgroups, LRR-II-1 and LRR-II-2, according to the presence or absence of members from *Arabidopsis* (Table 1, Figure S2). However, there were several branches without *Arabidopsis* members. *M*. *truncatula* and soybean clustered onto the branches of LRR-II-2 and LRR- III-2 specifically lacking *Arabidopsis* homologues (Table 1, Figure S2). LRR-II-2 comprised 16 members from *M*. *truncatula* and 18 from soybean, and LRR- III-2 was the smallest group, containing only one *M*. *truncatula* gene and 3 soybean members. These results suggested that they might be legume-specific clades. Some genes involved in nodulation symbiosis were clustered in the LRR-I and LRR-III-1 subgroups [69,74].

### 3.3. LRR-RLK Gene Structure and Conserved Motif Analysis

The structure of a gene largely determines its function. Numerous introns were already present in eukaryotic genes at the earliest stages of evolution of eukaryotes, and part of these sequences may be conserved in eukaryotes [75]. To study the genetic diversity of the MtLRR-RLK gene family, we analyzed the number and distribution of introns/exons and untranslated regions (UTRs) (Figure S3). Among all the MtLRR-RLK members identified in this study, the number of exons was ranged from 1–15, and Medtr5g030920 and Medtr8g059605 had the most exons (up to 15) (Table 2). Members with 14 exons accounted for 10.6%, and members with only one exon accounted for 3.9% (Figure S3). There were 172 members with more than 3 exons and 76 with more than 10 exons (Figure S3). By combining the results of our phylogenetic tree analysis with gene structure results, we found that gene members in the same group or subgroup have the similar the exon numbers. For instance, there were 38 members in LRR-III-1, with an average of 2.3 exons of and a maximum of 3 (Table 2). The LRR-XI-1 subgroup with the largest number of members had an average exon number of 2.5 and a maximum of only 7 (Table 2). The phylogenetic tree shows that the number of exons significantly differed in various groups, for example, the LRR-I and the LRR-II-2 subfamilies, although they clustered onto a large branch. These results are consistent with the results of phylogenetic tree analysis and increases the confidence to the classification of MtLRR-RL family.

Conserved domains are important regions for protein function. A typical LRR-RLK contains an LRR extracellular domain and an intracellular kinase domain [6]. Here, the type and number of motifs of the genes were predicted by using the program MEME. A total of 20 motifs were queried, and the size of the motifs ranged from 29–66 aa (Table S2). These results indicated that the type and the number of conserved motifs were similar between members in the same subgroup, such as LRR-II-1, LRR-IV, LRR-VI-1, LRR-VIII, and LRR-XII (Figure S3). In addition, the type and number of conserved motifs of the analogous protein between different subfamily members were somewhat different. In combination with the gene structure data, it was revealed that members with more exons had significantly fewer motif 14 (dark green motifs in the attachment) than do members with fewer exons/introns (Figure S3).

### 3.4. Genome Distribution Across Chromosomes and Tandem Duplication (TD) of Members of the MtLRR-RLK Gene Family

**Figure 2:**
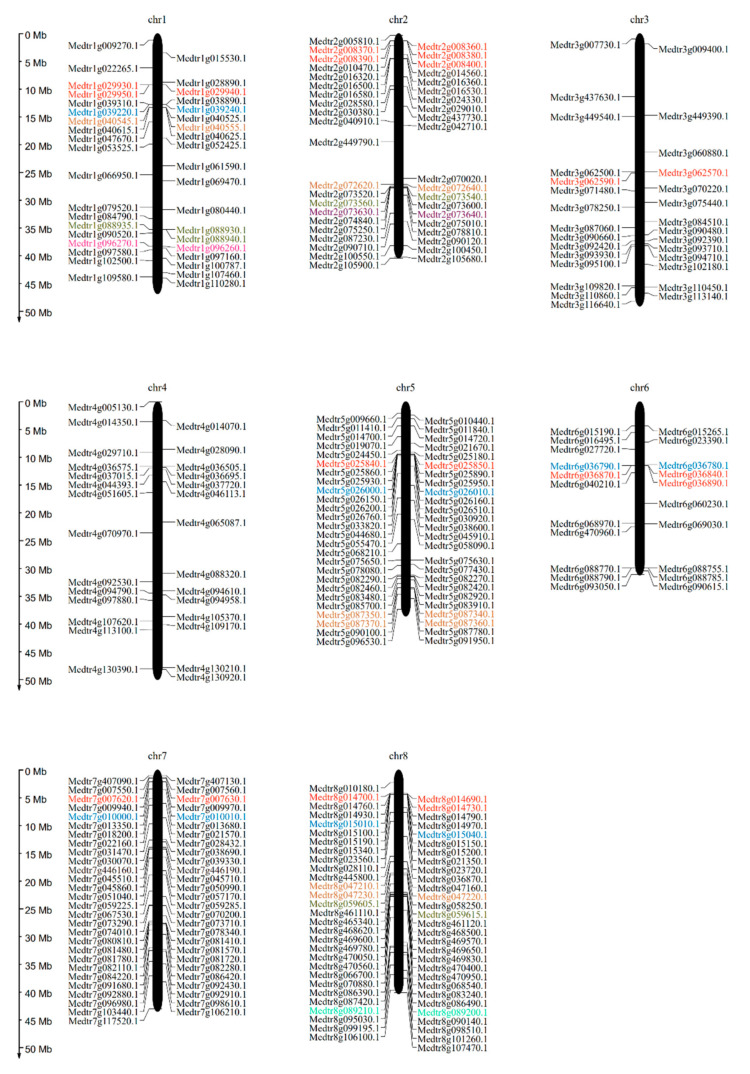
Genomic distribution of LRR-RLK genes across *M. truncatula* chromosomes. Chromosomal locations of *MtLRR-RLKs* were indicated based on the physical position of each gene. The positions of genes on each chromosome were drawn with Mapgene software and the number of chromosome was on the top of each chromosome. Genes with the same color represent a pair of tandem duplicated genes.

The position information of MtLRR-RLKs obtained from the genome database was used to map the genes onto corresponding chromosomes of *M*. *truncatula*. The results showed that 321 out of 329 *MtLRR-RLK* genes could be mapped onto all 8 chromosomes, while 8 members were localized to unassembled genomic sequence scaffolds and could not be mapped to any particular chromosome (Figure 2, Table S3). The overall distribution of members on the chromosomes was uneven—most of them were concentrated on chromosomes 1, 3, 5, 7, and 8, with a maximum of 58 on chromosome 8 and a minimum of 21 on chromosome 6 (Figure 2). Besides, to investigated the location of these 8 genes in chromosome, the position information obtained from the latest genome MtrunA17r5.0 database. It indicated that four, one and three genes were mapped onto chromosome 1, chromosome 4 and chromosome 8, respectively (Figure S4).

A gene family is a set of several similar genes formed by duplication of a single original gene, and the members generally have similar biochemical functions. An explosion in the number of members of a gene family has generally occurred as the result of repetitive TD and segmental and/or whole-genome duplication (S/WGD) events [76,77]. According to the descriptions of Holub [78], a chromosomal region within 200 kb containing two or more genes is defined as a TD event. We aligned protein sequences with MCScan to analyze the duplication events within the MtLRR-RLK family. Among all the MtLRR-RLK genes, a total of 23 TD gene pairs involving 56 genes were distributed across all chromosomes except chromosome 4; there were as many as 5 TD gene pairs involving 12 genes on chromosomes 1 and 8 (Table S4). Most TD clusters consisted of 2 genes, and the largest cluster had 5 tightly linked genes, which occurred on chromosome 2 (Figure 2). Most TD events were distributed in the LRR-XI-1 and LRR-XII subgroups, which were also the two subgroups with the most members (Table 1, Figure 2, Table S4). These results showed that TDs in *M. truncatula* are one of the reasons for the increase in the number of members of these two subgroups. Duplicated pairs of fragments located on the same chromosome were very close to each other on the chromosomes (Figure 2), as was the case for Medtr1g096260 & Medtr1g096270, Medtr8g047210 & Medtr8g047220. The sequence alignment showed that their sequences were similar, and loss or gain of various fragments can lead to the formation of functionally redundant genes.

### 3.5. Segmental Duplication and Synteny of the MtLRR-RLK Gene Family

**Figure 3:**
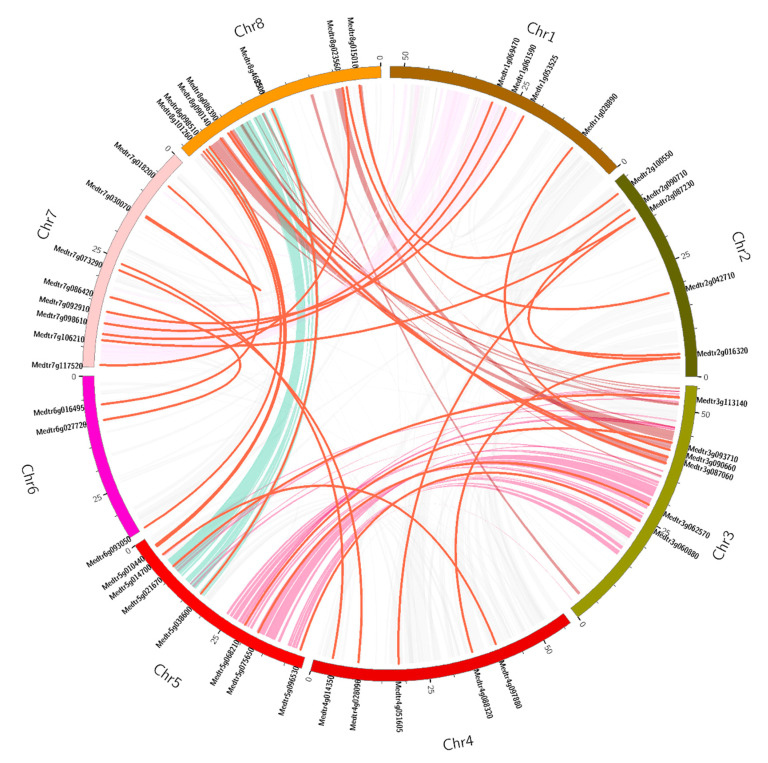
The Circos figure for chromosome locations with *MtLRR-RLK* segmental duplication links. The red lines indicate segmented duplicated gene pairs.

**Figure 4:**
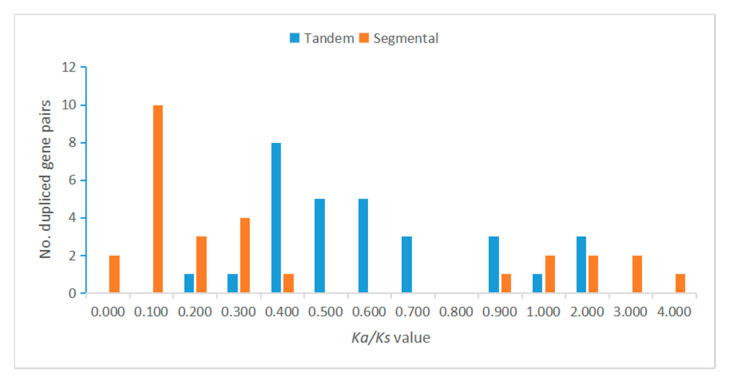
The distribution of *Ka/Ks* values in all tandem and segmental duplicated *MtLRR-RLKs*. The *Ka/Ks* value of each duplicated gene pair was calculated by KaKs_Calculator2.0.

**Figure 5:**
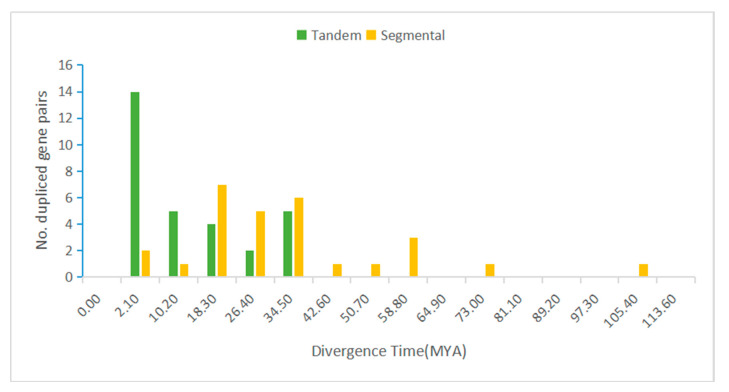
The distribution of Divergence Time (MYA) in all tandem and segmental duplicated *MtLRR-RLKs*. The divergence time of each duplicated gene pair was calculated by T = Ks/2γ (γ = 1.5 × 10^−8^). γ is the rate of divergence for nuclear genes of dicotyledonous plants.

Segmental duplications lead to duplicated genes through polyploidy, followed by chromosome rearrangements. We found that the *M. truncatula* genome contains segment replication events. Through MCScanX analysis, a total of 27 pairs of segmental duplications in the *MtLRR-RLK* gene family were found to be colinearly related, and for some segmental duplications, three LRR-RLK members were colinearly related (Figure 3, Table S4). The nonsynonymous (*Ka*) and synonymous (*Ks*) substitution rate values between these duplicated events were calculated via KaKs_Calculator. The *Ka/Ks* information is listed in Table S5. A *Ka/Ks* ratio equal to one indicates a ‘‘neutral mutation or no selection’’, and a *Ka/Ks* ratio less than one indicates ‘‘negative or purifying selection’’, whereas a *Ka/Ks* ratio greater than one indicates ‘‘positive or Darwinian selection’’ [79]. The distribution analysis of the *Ka/Ks* values suggested that all the *Ka/Ks* values of the tandem duplicated gene pairs ranged from 0 to 1.237, with one peak at 0.40–0.50, and all the *Ka/Ks* values of segmental duplicated gene pairs ranged from 0 to 3.582, with one peak at 0.10–0.20. There were 4 segmental event gene pairs in the range of 0.3 to 0.4 (Figure 4). Approximately 90% of the tandem duplicated gene pairs have a *Ka/Ks* value of less than 1, indicating that the selection pressure is negative (Figure 4). Moreover, we also calculated the divergence time (MYA) of duplication events. Forty-seven percent of the tandem duplicated gene pairs were determined to have occurred approximately 2.11 and 0.2 million years ago (MYA). In addition, a single TD event was calculated to occur at approximately 42.57 MYA. The date of segmental duplicated events suggested that all the approximate dates ranged from 4.44 to 113.27 MYA, with a peak at 18.30–26.40 and another at 34.50–42.60 MYA (Figure 5, Table S5).

**Figure 6:**
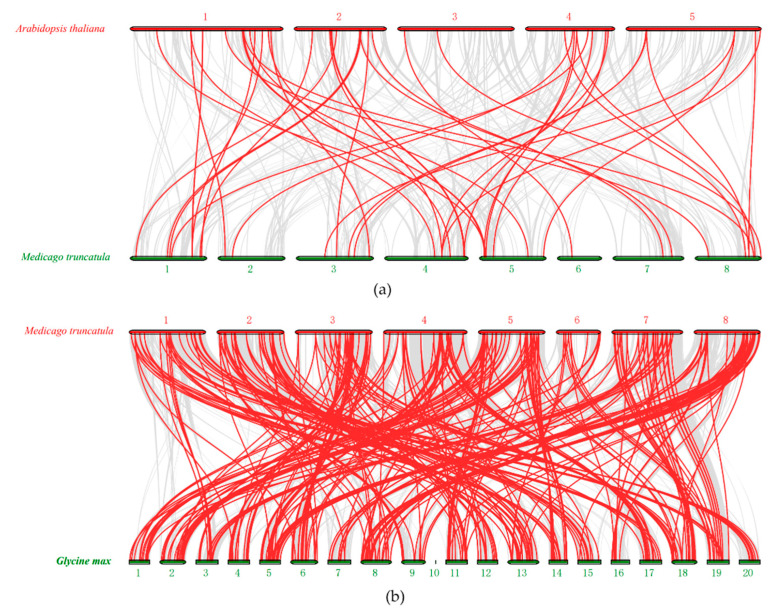
The synteny of LRR-RLK genes in different genomes of *M. truncatula*, *Arabidopsis* and soybean. (**a**) The synteny of *AtLRR-RLK* and *MtLRR-RLK* gene pairs. (**b**) The synteny of *GmLRR-RLK* and *MtLRR-RLK* gene pairs.

In addition, we analyzed the synteny of LRR-RLK genes between the *M*. *truncatula*, *Arabidopsis* and soybean genomes in this study. By comparing the whole genomes of *M*. *truncatula* and *Arabidopsis* via MCScanX to analyze the synteny between the two species, we identified a total of 43 gene pairs with synteny (Figure 6a, Table S6). More than 300 gene pairs were found to be collinearly related between *M*. *truncatula* and soybean (Figure 6b, Table S6). The collinear complexity of *M*. *truncatula* and soybean is much greater than that between *M*. *truncatula* and *Arabidopsis*. Together, these results indicated that *M*. *truncatula* and *Arabidopsis* were relatively distantly related while were closer with soybean; the genes of *M. truncatula* in the same family were more closely related to those of soybean than to those of *Arabidopsis*.

### 3.6. Prediction Analysis of Cis-Acting Elements within MtLRR-RLK Genes

To further study the potential regulatory mechanisms of *MtLRR-RLK* in growth and defense response processes, especially the response to biotic stress such as pathogen infection, 2.0 kb upstream sequences from the translation start sites of *MtLRR-RLK* genes were submitted to the PlantCARE database to detect cis-elements. Twenty elements, including abiotic stress-responsive elements, abscisic acid (ABA)-responsive elements, TC-rich repeats and W-boxes, were analyzed; these are shown in Figure S5. Within the 2.0 kb upstream sequence of the *MtLRR-RLK* members, in addition to the common TATA-box and basic cis-acting elements, most members contain ABRE elements, MBS elements, TATC-boxes, TGACG-motifs, GC-motifs, TC-rich motifs, W-boxes, G-boxes and other cis-elements related to biotic stress responses. By combining the results of our phylogenetic tree analysis with gene cis-elements results, we found that gene members in the same group or subgroup have the same cis-elements. But most cis-elements in MtLRR-RLK genes emerge a diversity distribution. Among them, AuxRR-cores (65), GARE-motifs (63), P-boxes (79), TGA-elements (201), and so forth, are involved in hormone responses, and W-boxes (192), G-boxes (245), and as-1 elements (213) are involved in the defense response against pathogens (Figure S5, Table S7). G-boxes and ACGTs compose a family of a class of cis-acting elements that respond to environmental factors; for example, the G-box and H-box elements within the promoter of *CHS15* in French bean are essential for floral and root-specific expression and as tissue-specific regulatory elements [80]. The as-1-like element or ocs element is also a type of plant defense response element that was originally isolated from the 35S promoter of the cauliflower mosaic virus and the nos and ocs gene promoters of *Agrobacterium tumefaciens* [81]. In the *MtLRR-RLK* family, most members contained G-boxes and as-1 elements (Figure S5).

### 3.7. Expression Patterns of MtLRR-RLK Genes in Different Tissues

**Figure 7:**
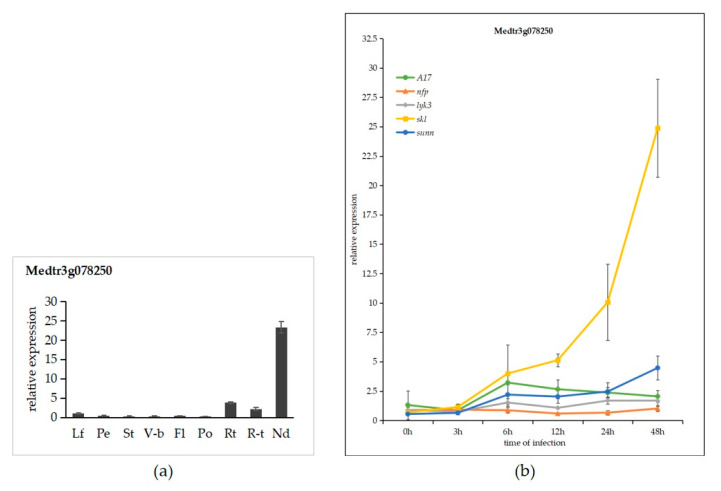
Expression profiles of MtLRR-RLK gene Medtr3g078250. (**a**) Expression levels of MtLRR-RLK gene Medtr3g078250 in various tissues. Expression profiles of Medtr3g078250 gene was analyzed in nine different tissues: leaf (L), petiole (Pe), stem (St), vegetablive bud (V-b), flower (Fl), pod (Po), root (Rt), root tip (R-t), nodule (Nd). The data are from three independent biological replicates, and error bars indicate standard deviations. (**b**) Expression of MtLRR-RLK gene Medtr3g078250 after *S. meliloti* infection in five *M. truncatula* genotypes: wild type A17, *nfp*, *lyk3*, *skl*, and *sunn* mutants. Values in the line graphs show average Trimmed Mean of M component (TMM) counts normalized to cv Jemalong A17 at 0 hpi. Error bars represent SE calculated from three independent biological replicates.

To gain a broader understanding of the putative functions of MtLRR-RLKs, we downloaded the RNA sequencing (RNA-seq) data within the *M*. *truncatula* PLEX Experiment Noble website ([68]; https://mtgea.noble.org/v3/). The expression of 189 LRR-RLK genes was determined in 12 different tissues, including flower, leaf, root, petiole, pod, seeding, stem, vegetative bud, and nodule tissue and at 3 different developmental times of the root nodules, from RNA-seq atlas data [68]. The results showed that most of them demonstrated distinct and inclusive tissue expression patterns in different tissues (Figure S6, Table S8). Detailed analysis showed that some of the members were expressed to varying degrees in every tissue—9 members were highly expressed in flowers, 17 were highly expressed in the leaves, 22 in the petioles, 24 in the pods, 42 in the stems, 29 in the vegetative buds, 36 in the seedlings, and 9 in the roots. More than one-third of the members are specifically expressed in the roots and nodules (Figure S6, A cluster with red box). Together, these findings suggest that these LRR-RLK genes might be involved in legume-rhizobium interactions and/or nodule development. Quantitative real-time PCR results indicated that the MtLRR-RLK gene Medtr2g078250 was highly expressed in roots and nodules, and specifically expressed in nodules, particularly (Figure 7a).

Detailed analysis also showed that some MtLRR-RLK tandem and segmental duplication gene pairs had similar expression patterns. For example, 6 out of 23 tandem duplicated gene pairs have very similar expression patterns—Medtr2g072620 and Medtr2g072640 as well as Medtr2g073540 and Medtr2g073560 are highly expressed in the roots and nodules specifically but hardly expressed in other tissues. Medtr7g010000 and Medtr7g010010 are expressed specifically in the leaves and petioles and highly expressed in leaves. Six out of 27 colinearly related segmental duplicated gene pairs also presented similar expression patterns. For example, Medtr1g061590 and Medtr7g103440 were expressed mainly in the pods, stems and shoots, indicating redundancy among them.

### 3.8. Expression Patterns of MtLRR-RLK Genes under Sinorhizobium Medicae Infection

To further explore the expression changes of the MtLRR-RLK genes in legume-rhizobium symbiosis under *S. medicae* infection, we used a database [69] (http://pages.discovery.wisc.edu/~sroy/Medicago_symbiosis_transcriptome/query.php) to identify a differential expression matrix of the corresponding members and construct an expression heatmap. In the treatment, there were three mutants and one wild type (WT) inoculated with *S*. *medicae*. A17 is a WT, with a normal nodulation phenotype. Mutants *nfp* [70] and *lyk3/hcl-1* [71] have no or decreased Nod factor sensitivities, respectively, while Nod factor-hypersensitive mutants (sickle, *skl* [72]) are supersensitive to *Rhizobium* and supernodulation [69]. For each mutant, transcriptional changes occurring in the roots of *M. truncatula* from 0, 0.5, 1, 3, 6, 12, 24, 36, and 48 h (a total of 9-time gradients) after inoculation with *Rhizobium* were acquired (Table S9). 

**Figure 8:**
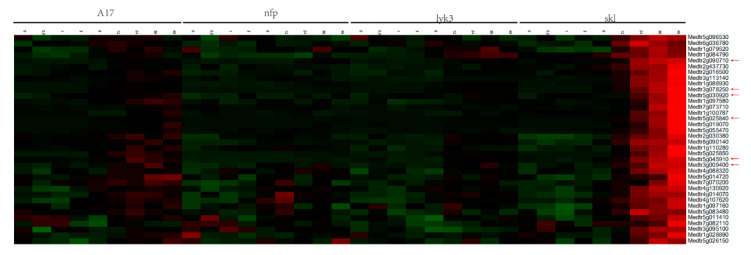
Expression profile of the 36 representative genes are highly expressed after rhizobium inoculation 12 h in mutant skl (http://pages.discovery.wisc.edu/~sroy/Medicago_symbiosis_transcriptome/query.php). Red arrows indicate genes that highly expressed in root, nodule and *skl* mutant under rhizobium infection.

The results of the expression levels showed that most of the members underwent transcription after 12 h of rhizobium inoculation (Figure S7), and a total of 36 members of MtLRR-RKLs (A the cluster with a purple box) was significantly highest after 12 h of rhizobium inoculation of the Nod factor-hypersensitive mutant *skl* (Figure 8, Figure S7). Moreover, their degree of expression was relatively high in WT, followed by *lyk3*, but hardly detected in *nfp*, which suggested that the expression of these genes was dependent on the Nod factor (Figure 8). Most of these members, especially Medtr5g030920 and Medtr3g078250, were located in the LRR-III and LRR-XI-1 subgroups (Table S10). These results indicated that these 36 gene members might be closely related to the symbiotic process of rhizobia and might play a certain function in regulating the biological process of nodulation induced by Nod factors. At the same time, they were highly expressed in *skl* mutant and the expression of 21 members peaked after 48 h of infection, which shown that they also specifically responded to ET signals during rhizobial infection [69]. While the expression of a small number of members (Figure S7, B cluster with a pink box) started to increase at 0.5 h after inoculation, after which their expression peaked but then decreased after 1–3 h. Most of these members that were highly expressed after 12 h and 0.5 h of rhizobium inoculation were in subgroups LRR-II and LRR-XI. The expression of some members (Figure S7, C cluster with a yellow box) peaked after 3 h of infection but then decreased; most of them were in subgroups LRR-I, LRR-II and LRR-VIII.

**Figure 9:**
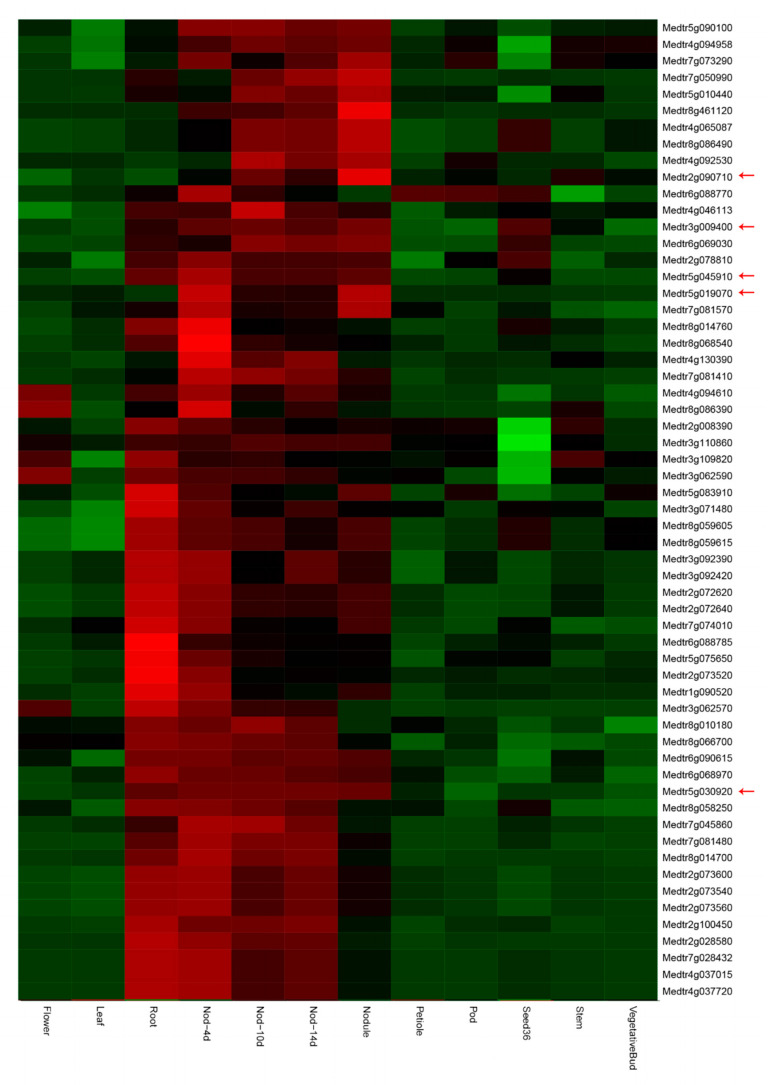
Organ expression patterns of the *MtLRR-RLKs* which are highly expressed in root and nodule (https://mtgea.noble.org/v3/). Red arrows indicate genes that highly expressed in root, nodule and *skl* mutant under rhizobium infection.

To identify the expression profile of these genes expressed specifically or highly in roots, nodules, and *skl* mutant after 12 h *S. meliloti* infection (Figure 8 and Figure 9), we determined the expression levels of one of the MtLRR-RLK genes (Figure 7). The expression level of Medtr3g078250 in *skl* mutant was much higher than in WT A17, *sunn* that involved in autoregulation of nodulation signal [73], *lyk3* and *nfp*, particularly in 12,24 and 48 hpi, which was similar with RNA-seq expression profile. In the *skl* mutants, the expression of Medtr3g078250, expressed specifically in root and nodules, was significantly increased starting 12 hpi. It was noted that the expression level in *sunn* was more equivalent or lower than that in WT A17 in 3,6,12,24 hpi, while it was obviously higher in 48 hpi (Figure 7b).

Combining the expression profiles in various tissues with those in the roots after *S*. *medicae* infection and qRT-PCR, we identified six gene members (Medtr5g030920/*DMI2*, Medtr2g090710, Medtr3g009400, Medtr3g078250, Medtr5g045910, Medtr5g019070) that were expressed preferentially in the roots and nodules and that responded specifically to Nod factors and ethylene (ET) signals in nodulation (Figure 7, Figure 8 and Figure 9). These genes are very likely related to symbiosis in *M*. *truncatula*. In addition, we found that the expression patterns of Medtr6g090615 and Medtr6g068970 were almost the same as that of Medtr5g030920/*DMI2* in the tissue expression profile, although these genes exhibited different expression dynamics in the roots after *Rhizobium* inoculation, with low expression in the *skl* mutant. The functions of these genes should be further verified. It was also found that the expression trends of 5 pairs of tandem gene pairs are similar. For example, Medtr5g025840 and Medtr5g025850 are highly expressed in the mutant *skl* after rhizobium inoculation, indicating that they may be functionally redundant. Notably, these genes clustered with the *Arabidopsis* EFR gene (AT5G20480) [43,82] in the phylogenetic tree; the EFR protein perceives bacterial EF-TU proteins and elicits an assortment of defense responses.

## 4. Discussion

Receptor-like protein kinases are ubiquitous in living organisms and function in receiving and transmitting information [2]. Compared with other eukaryotic kinase families, RLKs in *Arabidopsis* form a monophyletic gene family. The LRR-RLK gene family is one of the largest gene families of receptor kinases in plants. Its members are characterized by an extracellular domain that typically contains at least one leucine-rich repeats (LRR), a transmembrane domain, and an intracellular domain [83]. With respect to legume species, the LRR-RLK family in soybean has been identified, which has 467 members according to previous studies [45], and in this study, we identified a total of 329 members in *M. truncatula* (Table S1). There are at least 200 members in *Arabidopsis*, but the numbers differ in different studies [1,7,8,45]. Moreover, the current number of members in the LRR-RLK gene family is relatively large in various plant species, which also suggests that the LRR-RLK family is one of the largest members in plant RLK gene families [6,84]. According to the classification of *Arabidopsis* in our study, MtLRR-RLKs could be divided into 15 groups and 24 subgroups (Table 1). Among the 24 subgroups, the LRR-XI and LRR-XII subgroups had the most members. From the perspective of phylogenetic tree clustering, most members of *M*. *truncatula* correspond to one *Arabidopsis* member and two soybean members. There are also some members that clustered onto large branches within their own species. A similar phenomenon has also been found in soybean, rice and other plants, which reflects species specificity [12,44,45,46].

In our study, gene pairs with TDs were located in the same subgroup and were closely related. We found that most of the tandem duplicated genes in *M*. *truncatula* LRR-RLKs are located in subgroups LRR-XI and LRR-XII (Table S4), indicating that tandem duplication events are one of the main reasons for the expansion of these two subgroups in *M*. *truncatula*. A previous study on the evolutionary history of the *Arabidopsis* LRR-I subgroup discovered that an initial duplication gave rise to At1g51790 and the last common ancestor of the rest of the (ML-)*LRR-RLK* genes [85]. A second duplication gave rise to At1g51800 and the ancestor of the rest of the genes from groups LRR-I-1, LRR-I-2 and LRR-I-3 [85]. At1g51800 and At1g51790, as out-groups, seem to have been the genes to have emerged first from the cluster [85]. It was also suggested on the basis of the phylogenetic tree that *Arabidopsis* duplication events in the LRR-I subgroup resulted in an increase in the number of members. These results also proved that TD is one of the main ways that gene family members evolve and expand [86,87,88]. The duplication and collinearity analysis of the MtLRR-RLK family showed that genes involved in TD or collinearity are highly similar in terms of phylogenetic evolution, gene size, gene structure, conserved motifs, and so forth, but lack a certain difference in expression patterns. Compared with tandem repeat genes, collinear genes were located mostly on different chromosomes or on the same chromosome but in different regions.

Analysis of the expression data revealed that genes in the same subgroup have similarities. The close phylogenetic and expression profiling analysis between MtLRR-RLK genes and *Arabidopsis* or soybean genes in the same group also provides insight into their putative functions. With respect to the expression pattern of the Nod factor-hypersensitive mutant *skl* under rhizobial infection, we seemed to find that at least 36 gene members (Table S10), including DMI2, were highly expressed specifically after rhizobium inoculation. Among these genes, six were also highly expressed in the roots and during nodule development of *skl* mutants. This implied that these genes may be good candidates to regulate nodulation (Figure 7, Figure 8 and Figure 9). *skl* is also an ET-insensitive mutant, suggesting that Nod factor signaling activates ET production to attenuate its own signal [69].

We know that some *Arabidopsis* LRR-RLK functions have been resolved in previous research. For example, AT1G51790, a member of the LRR-I group, is involved in downy mildew disease [85]. MtDMI2, which is in the same group as AT1G51790, is one of the major receptors in the Nod factor signaling pathway in *M. truncatula* [54,89]. In the LRR-III group, several *Arabidopsis* members, such as transmembrane kinase-like (TMKL1), RKL1, and PXY-correlated 1(PXC1), have important functions in secondary wall formation, silique maturation, and organ/tissue development [90,91,92]. In this study, 9 members, including Medtr7g073710 and Medtr3g078250, whose expression was induced specifically by rhizobial infection (Table S10), also clustered in the LRR-III group. However, they were not on the same branch and may play totally different roles in plant development. The *Arabidopsis Strubbelig-receptor Family* (*SRF*) gene family members, which are in the LRR-V group, affect different aspects of cell wall biology [93]. Some members of the LRR-XV group are involved in *Arabidopsis* transmembrane signaling [94,95]. The BAK1-interacting Receptor 1 (BIR1) and BRI1 genes are involved in BR and peptide signaling in the LRR-Xa and LRR-Xb groups, respectively [25,26,30]. The extracellular domain of BRI1 binds to BRs [24]. BRI1 then interacts with another AtLRR-RLK BAK1 and functions together to mediate plant steroid signaling [25]. Another important group is the LRR-XI groups, in which CLV1 and HAESA (formerly named RLK5) control the size of the apical meristem, floral meristem, and floral organ abscission [31,96]. Interestingly, 8 members (including Medtr1g097580, Medtr2g090710, Medtr2g437730, Medtr5g045910 and Medtr5g014720) whose expression was induced specifically by Nod factors also grouped into LRR-XI (Table S10). SUNN (Medtr4g070970), an important CLV1-like gene involved in autoregulation of the nodulation signaling pathway in *M. truncatula* [97], was also in the LRR-XI group within the same clade as AtCLV1 (Figure S2). Group LRR-XII is represented by *Arabidopsis* LRR-RLKs with important roles in the perception of bacterial pathogens, such as FLS2 and EFR [41,42,43]. In the LRR-XII subgroup, 6 genes, including the TD gene pair Medtr5g025840 and Medtr5g025850, were expressed specifically under rhizobial infection (Table S10) and clustered onto the same branch containing AtEFR, and there are several possible similarities in their functions. Group LRR-XIII members clustered with ERECTA, which regulates cell wall-mediated resistance to pathogens [28,98,99].

Complex biological processes may involve the recruitment of different LRR-RLKs to participate. For example, the functions of DMI2 (Medtr5g030920) and SUNN (Medtr4g070970), which are members of the LRR-I and LRR-XI groups, have been specifically studied in the regulation of symbiosis, but their own mechanisms are different [51,54,97]. The MtDMI2 protein functions as a coreceptor of rhizobial signals to initiate nodule development and rhizobial infection during nitrogen fixation symbiosis according to the extracellular region of two malectin-like domains (MLDs) [89]. It was specifically expressed in the roots and nodules according to the tissue expression profile and *S. meliloti* infection (Figure 8 and Figure 9). SUNN, a CLV1-like LRR-RLK, regulates nodule number and root length and is expressed in the roots, flowers and shoots of *M. truncatula,* and it was suggested that four *sung* mutants (*sung-1* to *sung-4*) have shortened roots and experience hypernodulation [97]. Interestingly, we found that the members of LRR-XI group of the LRR-RLKs of the three species could be divided into three large clades—a CLV1-like representative branch, an RGF1 INSENSITIVE (RGI) representative branch and a third clade (Figure S2). In *Arabidopsis*, CLV1 controls stem cell number and differentiation at the shoot and flower meristems [31,100]. The expression of another CLV1-like MtLRR-RLK, Nodule-induced Receptor-Like Kinase 1 (MtNRLK1; Medtr5g090100), was upregulated during nodulation [101]. MtNRLK1 clustered onto the same branch as did SUNN. GmLRK1, GmCLV1B/NARK, GmCLV1A, and GmFON1, with known functions in soybean, were also members of the LRR-XI group and are closely related to AtCLV1 [102,103,104]. AtRGI, one of the members on the second branch in LRR-XI, did not cluster with AtCLV1 in the same clade. RGF1 INSENSITIVE 1 to 5 (AtRGI1-5; At3G24240, At5G48940, At4G26540, At5G56040, AT1G34110) play essential roles in RGF1-PLT-mediated root meristem development in *Arabidopsis* [37]. Among the MtLRR-RLKs, Medtr5g045910, Medtr7g059285, Medtr3g060880, Medtr4g105370 and Medtr1g097580 are homologues of RGI1-5 in Arabidopsis, and their functions may be similar to that of AtRGI; Medtr5g045910 and Medtr1g097580 were expressed preferentially in the roots and nodules and were induced specifically in response to *S. meliloti* inoculation (Figure 8 and Figure 9). Therefore, these genes may play a particular role in the development of roots and nodules in *M. truncatula*. Moreover, Medtr5g045910, Medtr5g019070 and Medtr5g025840, which are members of groups LRR-XI and LRR-XII respectively, have a major effect on downstream symbiotic gene expression and are highly associated with known symbiotic genes [69]. In addition, the expression profile showed that Medtr3g078250 also specifically responded to Nod factors and ET signals during rhizobial infection (Figure 7 and Figure 8). Other studies have proposed that homologue of Medtr3g078250, *Rhizobial Infection Receptor-like Kinase 1* (*LjRINRK1*), is an infection-specific RLK and may specifically coordinate output from Nod factor signaling or perceive an unknown signal required for rhizobial infection [74].

In both of animals and plants, LRR-RLKs intervene plenty of signaling messages at the cell surface and act as key regulators during development processes. As receptors on the cell surface, MtLRR-RLKs have important functions in the sensing and receiving of signals from their surrounding environment to regulate internal responses. Moreover, symbiosis is a specific and necessary function in legume plants, it suggests that MtLRR-RLKs participate in controlling both Nod factor signal transduction and endomycorrhizae formation. The combination of the expression profile data and the results of various bioformatic analyses in this study suggested that at least 36 members of MtLRR-RLKs play a potential role in nodulation and three AtRGI homologues in regulating meristem and organ development. Our results provide a framework information for the *LRR-RLK* gene family in *M. truncatula* and serve as a guide for functional research of the *LRR-RLKs*, especially in nodulations and symbioses. However, expression profile data of MtLRR-RLKs we obtained and analyzed just is the part of *M. truncatula* genome transcription studies. The expression of some MtLRR-RLK genes that may not be fully investigated might have analogous and important functions in symbiosis. The specific functions of these promising MtLRR-RLKs should be further researched in the future.

## 5. Conclusions

In this study, a genome-wide analysis of the *M. truncatula* LRR-RLK family was performed, and a total of 329 putative LRR-RLKs were confirmed. They were classified into 15 groups of 24 subgroups according to the classification of *Arabidopsis*. Our analyses of the *MtLRR-RLK* genes of *M. truncatula, Arabidopsis and soybean* revealed their diversity in terms of member number, phylogeny, gene structure, chromosomal location, gene duplication, cis-acting elements, tissue expression patterns and expression patterns in response to symbiosis. The expression patterns in in various tissues and under rhizobial infection suggest that there may be some candidate MtLRR-RLK family receptor members that play a specific role in the signal transmission and transduction process and development during symbiosis. This study provides comprehensive information on the *LRR-RLK* gene family in *M. truncatula*, and efforts to identify specific gene functions of the *MtLRR-RLK* genes will be conducted in the future.

## Figures and Tables

**Table 1:** List of leucine-rich repeat receptor-like kinase (LRR-RLK) subfamily names and numbers of *Arabidopsis*, *M. truncatula* and soybean.

LRR-RLK Subfamily	*Arabidopsis*	*M. truncatula*	Soybean
RLK-Pelle_LRR-I	50	23	23
RLK-Pelle_LRR-II-1	14	14	26
RLK-Pelle_LRR-II-2	0	16	18
RLK-Pelle_LRR-III-1	46	38	87
RLK-Pelle_LRR-III-2	0	1	3
RLK-Pelle_LRR-IV	3	4	8
RLK-Pelle_LRR-V	9	8	18
RLK-Pelle_LRR-VI-1	5	6	13
RLK-Pelle_LRR-VI-2	8	7	16
RLK-Pelle_LRR-VII-1	5	5	10
RLK-Pelle_LRR-VII-2	3	3	7
RLK-Pelle_LRR-VII-3	2	2	6
RLK-Pelle_LRR-VIII	8	7	17
RLK-Pelle_LRR-IX	4	11	15
RLK-Pelle_LRR-Xa	4	3	5
RLK-Pelle_LRR-Xb-1	9	5	15
RLK-Pelle_LRR-Xb-2	1	2	4
RLK-Pelle_LRR-XI-1	33	99	123
RLK-Pelle_LRR-XI-2	2	1	2
RLK-Pelle_LRR-XII	8	65	28
RLK-Pelle_LRR-XIIIa	4	2	6
RLK-Pelle_LRR-XIIIb	3	2	8
RLK-Pelle_LRR-XIV	2	2	4
RLK-Pelle_LRR-XV	2	3	5
Total numbers	225	329	467

**Table 2:** List of the exon number of LRR-RLKs subfamilies in *M. truncatula.*

LRR-RLK Subfamily	Number of Genes	Maximum Number of Exons	Minimum Number of Exons	Average Number of Exons	Standard Deviation
RLK-Pelle_LRR-I	23	15	9	12.87	1.14
RLK-Pelle_LRR-II-1	14	12	10	11.00	0.39
RLK-Pelle_LRR-II-2	16	15	14	14.06	0.25
RLK-Pelle_LRR-III-1	38	3	2	2.29	0.46
RLK-Pelle_LRR-III-2	1	2	2	2.00	0
RLK-Pelle_LRR-IV	4	5	4	4.25	0.5
RLK-Pelle_LRR-V	8	14	14	14.00	0
RLK-Pelle_LRR-VI-1	6	7	6	6.83	0.41
RLK-Pelle_LRR-VI-2	7	13	9	11.57	1.09
RLK-Pelle_LRR-VII-1	5	2	2	2.00	0
RLK-Pelle_LRR-VII-2	3	2	1	1.67	0.58
RLK-Pelle_LRR-VII-3	2	1	1	1.00	0
RLK-Pelle_LRR-VIII	7	14	14	14.00	0
RLK-Pelle_LRR-IX	11	6	2	2.40	1.26
RLK-Pelle_LRR-Xa	3	2	2	2.00	0
RLK-Pelle_LRR-Xb-1	5	1	1	1.00	0
RLK-Pelle_LRR-Xb-2	2	4	2	3.00	1
RLK-Pelle_LRR-XI-1	99	7	1	2.49	1.18
RLK-Pelle_LRR-XI-2	1	1	1	1.00	0
RLK-Pelle_LRR-XII	65	7	2	3.19	1.28
RLK-Pelle_LRR-XIIIa	2	13	13	13.00	0
RLK-Pelle_LRR-XIIIb	2	14	14	14.00	0
RLK-Pelle_LRR-XIV	2	4	4	4.00	0
RLK-Pelle_LRR-XV	3	1	1	1.00	0
Total	329	165	131	144.62	

## Supplementary Materials

The following are available online at https://www.mdpi.com/2075-1729/10/9/176/s1, Figure S1: Unrooted phylogenetic tree of MtLRR-RLKs. The coding sequences from 329 MtLRR-RLKs were aligned by Clustal W and the phylogenetic tree was constructed using the MEGA 7.0 by the neighbor-joining with 1000 bootstrap replicates. Figure S2: Unrooted phylogenetic tree of MtLRR-RLKs, GmLRR-RLKs and AtLRR-RLKs. The coding sequences from 329 MtLRR-RLKs,467 GmLRR-RLKs and 225 AtLRR-RLKs were aligned by Muscle and the phylogenetic tree was constructed using the MEGA 7.0 by the Neighbor-joining with 1000 bootstrap replicates. Figure S3: The exon/intron, UTR organization and motifs of all MtLRR-RLK genes. Exons are represented by blue boxes and introns by black lines. UTR regions of some genes are also indicated using red boxes. The relative sizes of exons, introns and UTR can be estimated by the length of boxes or lines. 20 motifs were predicted. Figure S4: Genomic distribution of LRR-RLK genes across *Medicago truncatula* chromosomes including 8 genes non-mapped on v4 genome. The position of eight genes that were localized to unassembled genomic sequence scaffolds in v4.0 genome are acquired in A17 MtrunA17r5.0 genome. Figure S5: The predicted cis-acting element in *MtLRR-RLKs* promoters. The 2.0 kb upstream sequences of the *MtLRR-RLKs*-genomic DNA sequences were retrieved. Figure S6: The heatmap of expression for *MtLRR-RLK* genes across different tissues. The genome-wide RNA-seq data of *M. truncatula* were obtained from *M. truncatula* PLEX Experiment Data. The expression data of MtLRR-RLKs in flower, leaf, root, nod-4, nod-10, nod-14, nodule, petiole, pod, seed36, stem, vegetative bud was genewise normalized and hierarchically clustered. The color scale below represents expression values, green indicating low levels while red indicating high levels of transcript abundance (https://mtgea.noble.org/v3/). Figure S7: The heatmap of expression for *MtLRR-RLKs* under rhizobium infection. The genome-wide RNA-seq data of soybean were obtained from Query genes expression website. The expression data of MtLRR-RLKs in WT A17 and mutants with absent or decreased Nod factor sensitivities (i.e., Nodulation factor perception (*nfp*) and lysine motif domain-containing receptor-like kinase 3 (*lyk3*), respectively) and Nod factor-hypersensitive mutant (sickle, *skl*). This data set encompasses nine time points, allowing observation of the symbiotic regulation of diverse biological processes with high temporal resolution (http://pages.discovery.wisc.edu/~sroy/Medicago_symbiosis_transcriptome/query.php). Table S1: List of the identified LRR-RLK genes in *M. truncatula*. The ID, gene code, gene length, physical position on chromosome, number of exon/intron/UTR, length of amino-acid, number of kinase domain for each MtLRR-RLK gene in this study were included. Table S2: Putative motifs of all MtLRR-RLKs in each subgroup predicted by MEME. Motifs were identified by MEME software using the deduced amino-acid sequences of MtLRR-RLKs in each group and the relative position of each identified motif was shown. Table S3: The physical chromosomal positions of *MtLRR-RLKs* genes and chromosome length of M. *truncatula*. The position of eight were localized to unassembled genomic sequence scaffolds in v4.0 genome data are acquired in A17 MtrunA17r5.0 in website (http://plantgrn.noble.org/LegumeIP/gdp/25/gene). Table S4: List of tandem and segmental duplicated LRR-RLK gene pairs in M. *truncatula*. A region containing two or more MtLRR-RLK genes within 200 kb was defined as a tandem duplication cluster. Table S5: List of *Ka/Ks* values and divergence time of LRR-RLK tandem and segmental gene pairs in *M. truncatula*. Table S6: List of synteny gene pairs in *M*. *truncatula* and *Aradopsis*, *M. truncatula* and soybean genome. Table S7: The data of cis-acting element in *MtLRR-RLKs* promoters. Table S8: Expression profiles for *MtLRR-RLKs* across different tissues (https://mtgea.noble.org/v3/). Table S9: Expression profiles for *MtLRR-RLKs* under rhizobium infection (http://pages.discovery.wisc.edu/~sroy/Medicago_symbiosis_transcriptome/query.php). Table S10: List of *MtLRR-RLKs* high specifically expressed under rhizobium infection 12 h. Members in yellow shading were specifically expressed in roots, nodule and *skl* mutant. (http://pages.discovery.wisc.edu/~sroy/Medicago_symbiosis_transcriptome/query.php). Table S11: The primers used for quantitative real time RT-PCR.
