# Supplementary material for: Genome-Wide Characterization, Evolution, and Expression Analysis of the Leucine-Rich Repeat Receptor-Like Protein Kinase (LRR-RLK) Gene Family in Medicago truncatula
Source: Life (Basel). 2020 Sep 4;10(9):176. doi: 10.3390/life10090176 (PMC7555646; doi:10.3390/life10090176)
Supplement: Supplementary file 1 [file life-10-00176-s001.zip › Supplementary file/FigureS1 Unrooted phylogenetic tree of MtLRR_RLKs.pdf]

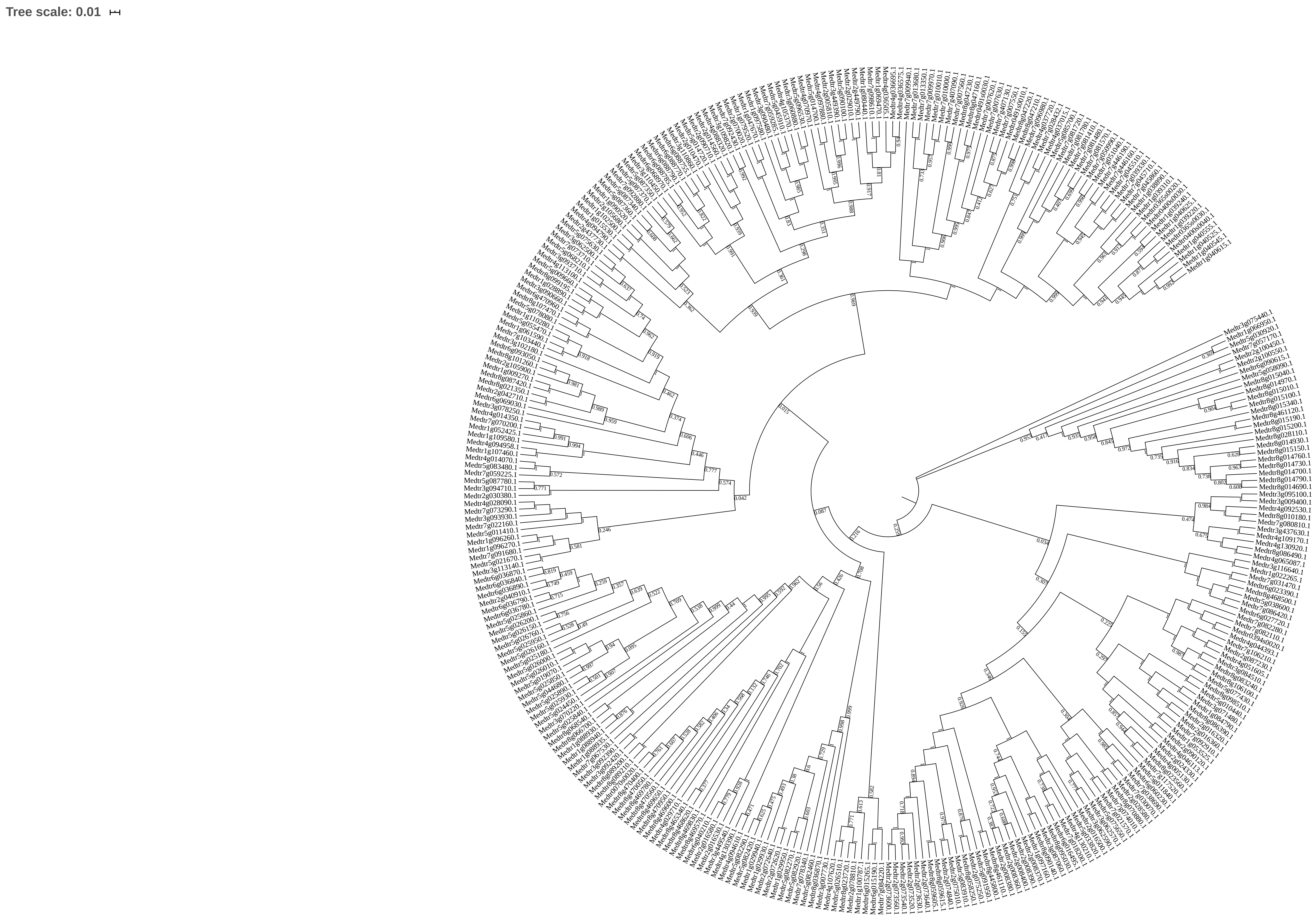
Tree scale: 0.01
