# Supplementary figures and images for: Genome-Wide Characterization, Evolution, and Expression Analysis of the Leucine-Rich Repeat Receptor-Like Protein Kinase (LRR-RLK) Gene Family in Medicago truncatula
Source: Life (Basel). 2020 Sep 4;10(9):176. doi: 10.3390/life10090176 (PMC7555646; doi:10.3390/life10090176)

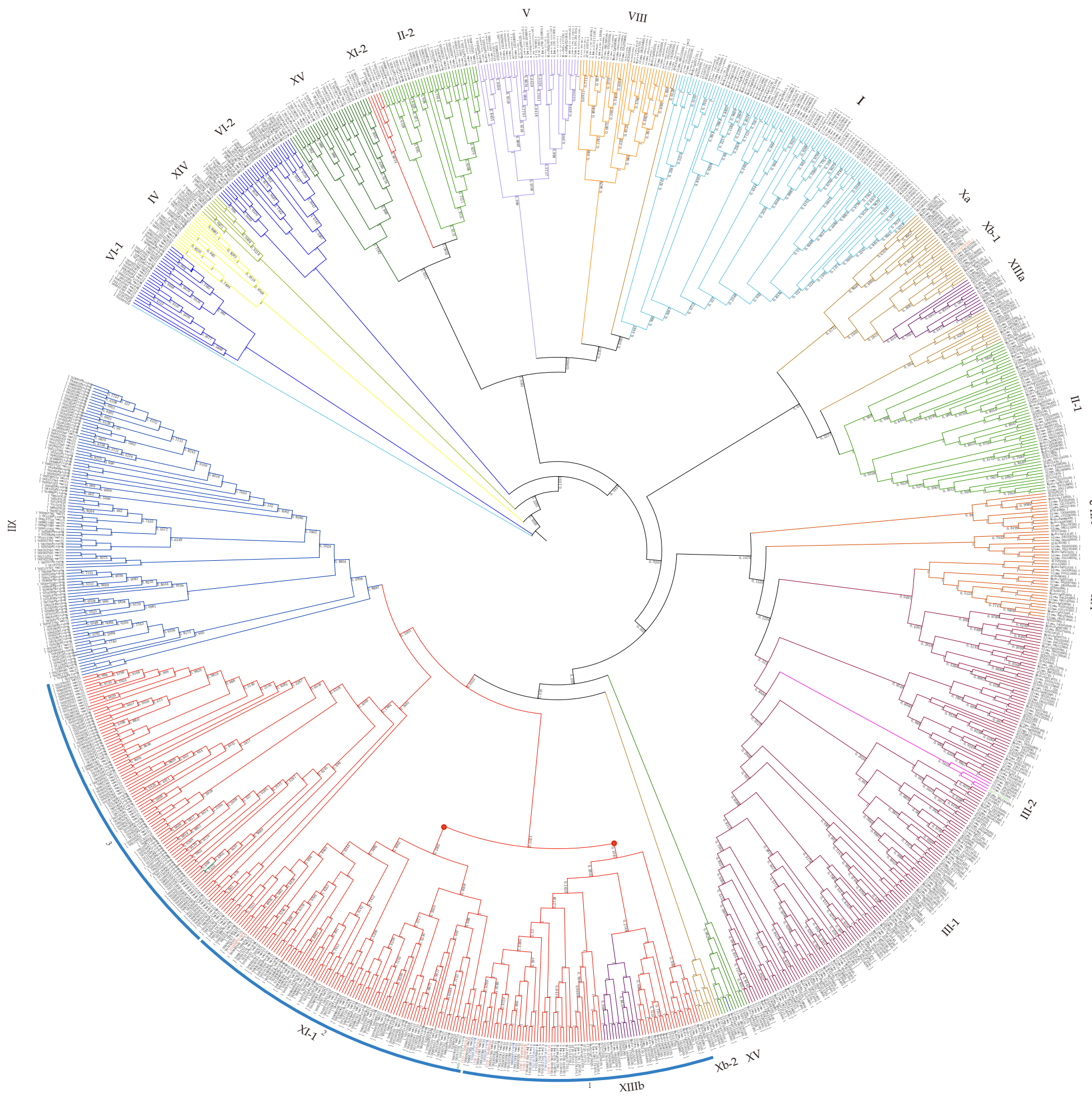

Supplement: Supplementary file 1 [file life-10-00176-s001.zip › Supplementary file/FigureS2 Unrooted phylogenetic tree of MtLRR-RLKs, GmLRR-RLKs and AtLRR-RLKs.pdf]

chr1

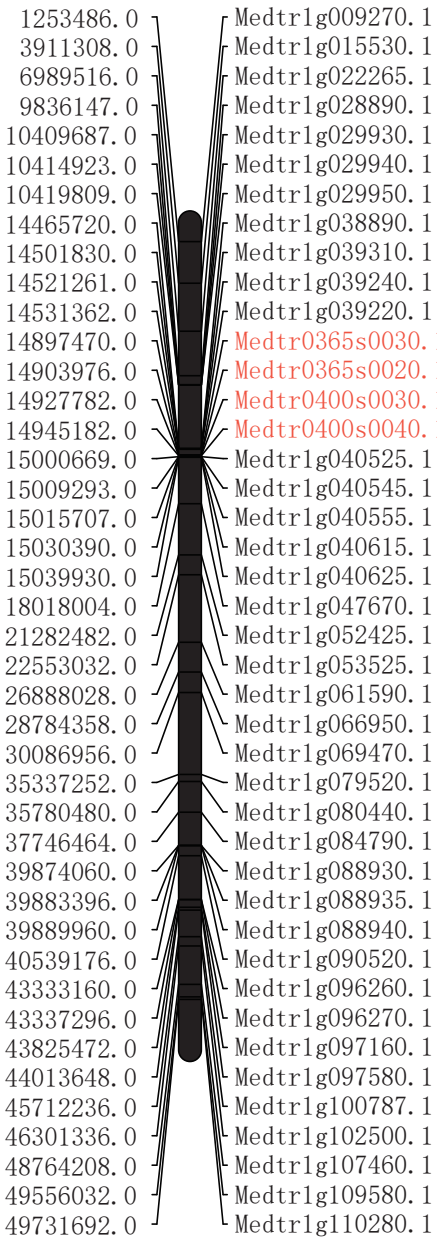

chr2

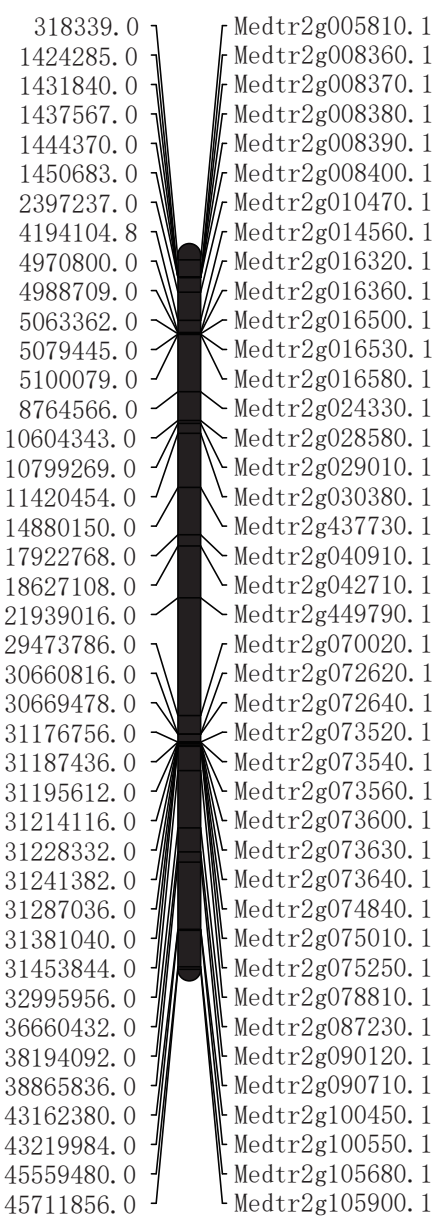

chr3

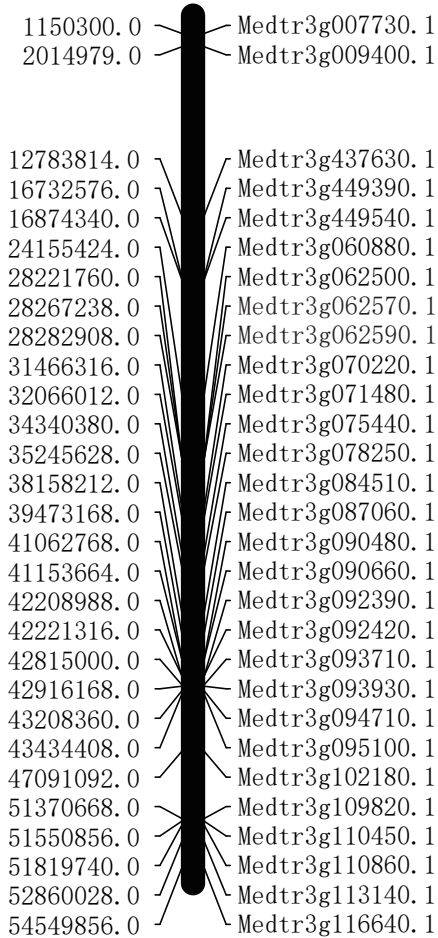

chr4

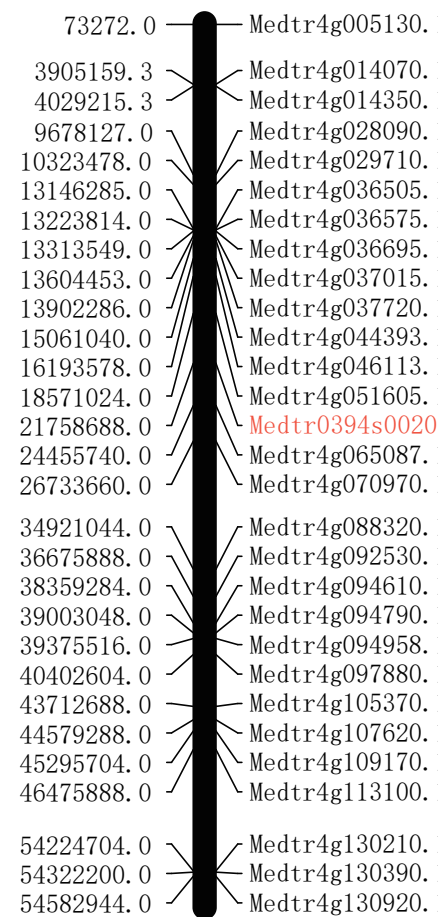

chr5

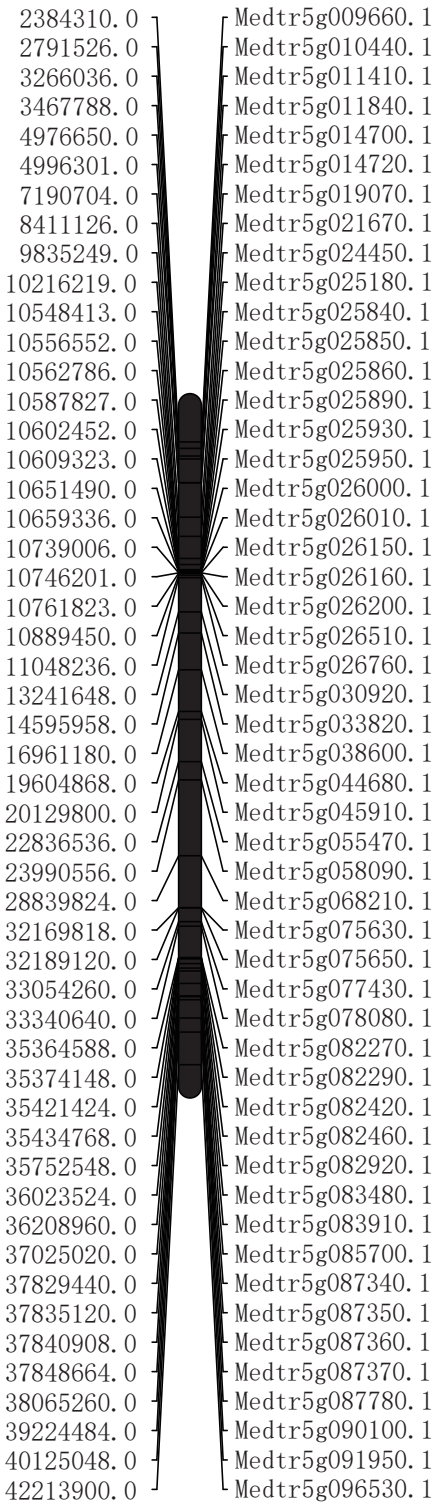

chr6

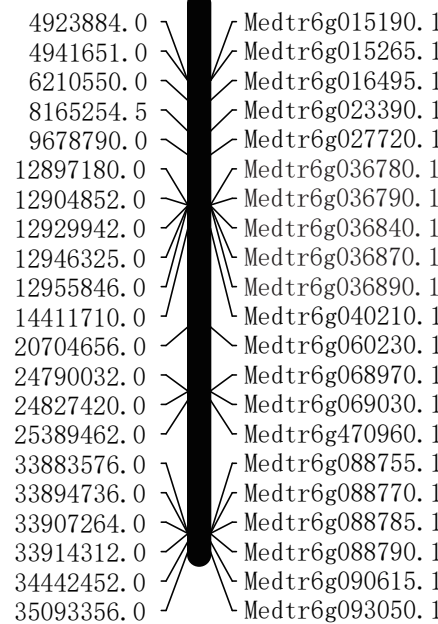

chr7

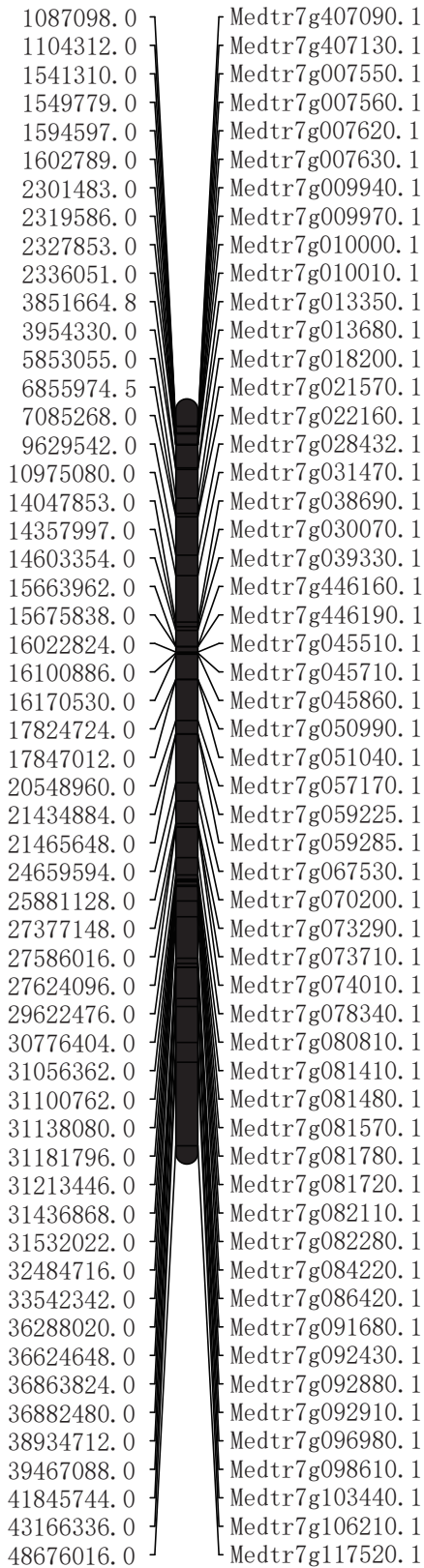

chr8

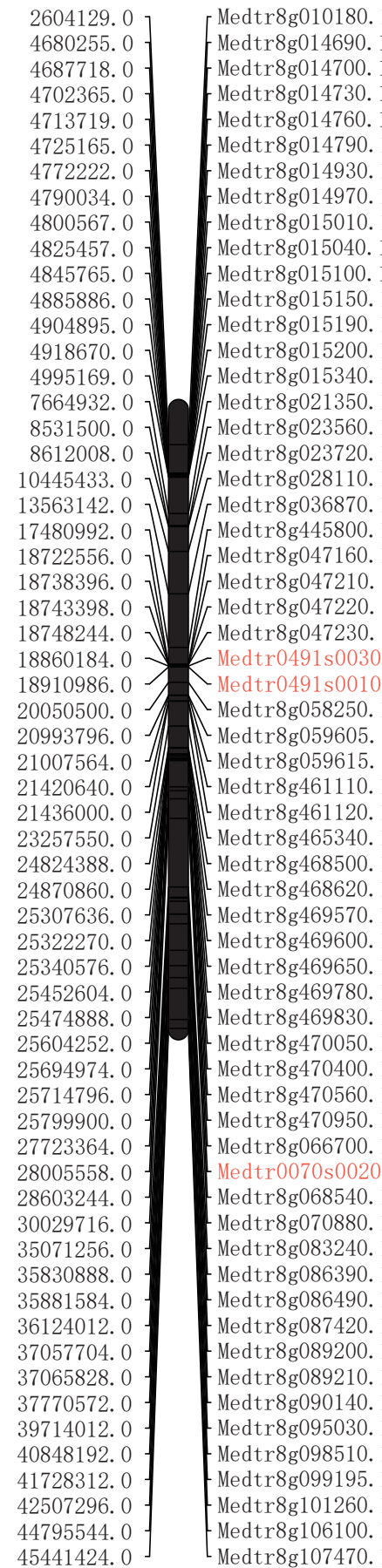

Supplement: Supplementary file 1 [file life-10-00176-s001.zip › Supplementary file/FigureS4 Genomic distribution of LRR-RLK genes across Medicago truncatula chromosomes including 8 genes non-mapped on v4 genome.pdf]

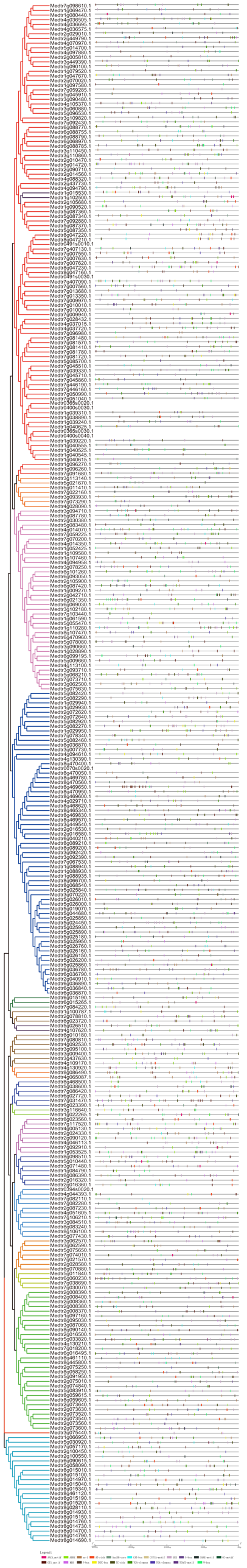

Supplement: Supplementary file 1 [file life-10-00176-s001.zip › Supplementary file/FigureS5 The predicted cis-acting element in MtLRR-RLK genes promoters.pdf]

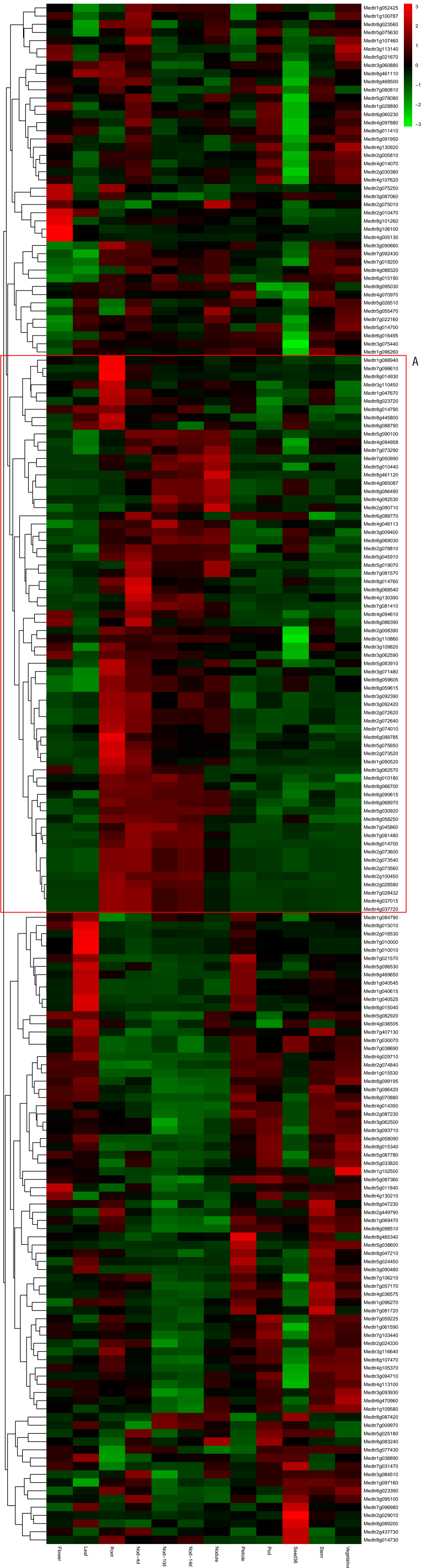

A

Supplement: Supplementary file 1 [file life-10-00176-s001.zip › Supplementary file/FigureS6 The heatmap of expression for MtLRR-RLK genes across different tissues.pdf]

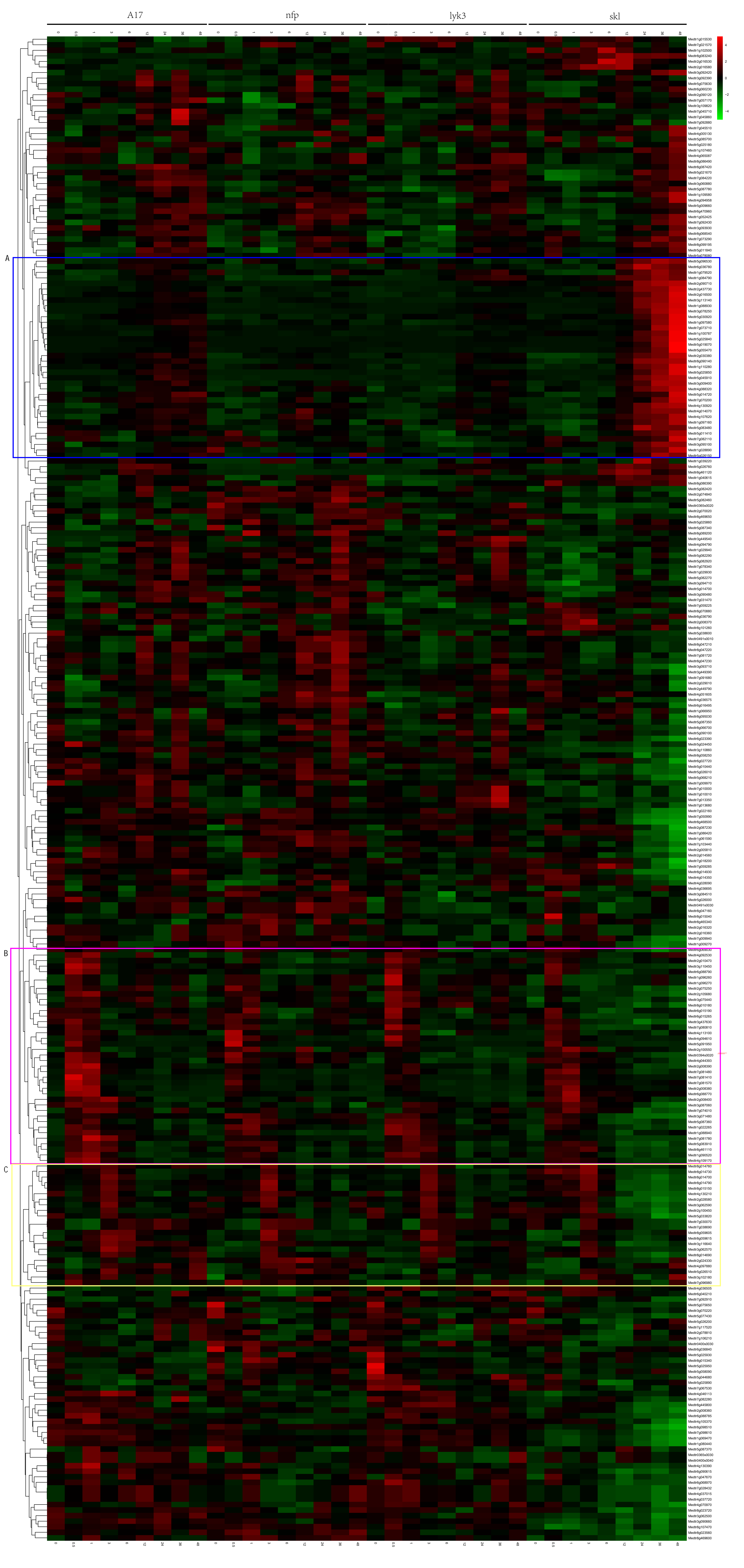

Supplement: Supplementary file 1 [file life-10-00176-s001.zip › Supplementary file/FigureS7 The heatmap of expression for MtLRR-RLK genes under rhizobium infection.pdf]
