# Supplementary material for: Genome-Wide Characterization, Evolution, and Expression Analysis of the Leucine-Rich Repeat Receptor-Like Protein Kinase (LRR-RLK) Gene Family in Medicago truncatula
Source: Life (Basel). 2020 Sep 4;10(9):176. doi: 10.3390/life10090176 (PMC7555646; doi:10.3390/life10090176)
Supplement: Supplementary file 1 [file FigureS3 The exon,intron, UTR organization and motifs of all MtLRR-RLK genes.pdf › Supplementary file/FigureS3 The exon,intron, UTR organization and motifs of all MtLRR-RLK genes.pdf]

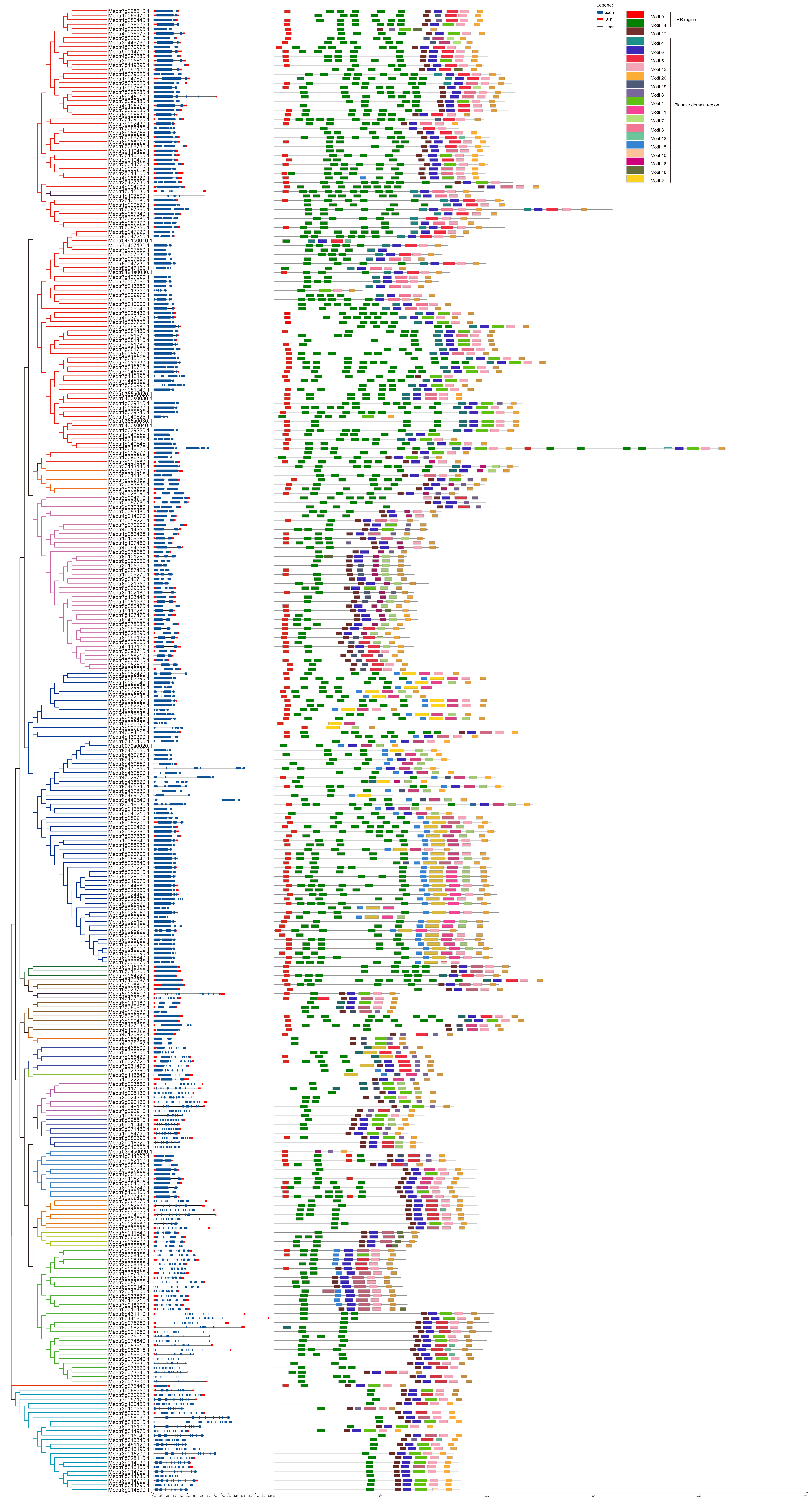

Legend:
exon
UTR
Intron
Motif 9
Motif 14
Motif 17
LRR region
Motif 4
Motif 6
Motif 5
Motif 12
Motif 20
Motif 19
Motif 8
Motif 1
Motif 11
Motif 7
Motif 3
Motif 13
Motif 15
Motif 10
Motif 16
Motif 18
Motif 2
Pkinase domain region
